# Potential productivity loss from uncorrected and under-corrected presbyopia in low- and middle-income countries: A life table modeling study

**DOI:** 10.3389/fpubh.2022.983423

**Published:** 2022-10-11

**Authors:** Qian Ma, Min Chen, Dehua Li, Ruiqing Zhou, Yali Du, Shengjie Yin, Binyao Chen, Hongxi Wang, Jiao Jiang, Zhiqiang Guan, Kunliang Qiu

**Affiliations:** Joint Shantou International Eye Center of Shantou University and the Chinese University of Hong Kong, Shantou, Guangdong, China

**Keywords:** potential productivity loss, life table modeling, productivity-adjusted life years, presbyopia, low- and middle-income countries (LMICs)

## Abstract

**Objective:**

To estimate the burden of potential productivity losses due to uncorrected and under-corrected presbyopia in LMICs among the working-age population in both the cross-sectional and longitudinal manner.

**Methods:**

We extracted data for the prevalence of presbyopia from the Global Burden of Diseases (GBD), Injuries, and Risk Factors Study 2019. Data for the gross domestic product (GDP) per capita were extracted from the World Bank database and Central Intelligence Agency's World Factbook. We introduced life table models to construct age cohorts (in 5-year age groups) of the working-age population (aged from 40 to 64 years old) in LMICs, with simulated follow-up until 65 years old in people with and without uncorrected presbyopia. The differences in productivity-adjusted life years (PALYs) lived and productivity between these two cohorts were calculated. The potential productivity loss was estimated based on GDP per capita. The WHO standard 3% annual discount rate was applied to all years of life and PALYs lived.

**Results:**

In 2019, there were 238.40 million (95% confidence interval [CI]: 150.92–346.78 million) uncorrected and under-corrected presbyopia cases in LMICs, resulting in 54.13 billion (current US dollars) (95% confidence interval [CI]: 34.34–79.02 billion) potential productivity losses. With simulated follow-up until retirement, those with uncorrected and under-corrected presbyopia were predicted to experience an additional loss of 155 million PALYs (an average loss of 0.7 PALYs per case), which was equivalent to a total loss of US$ 315 billion (an average loss of US$ 1453.72 per person).

**Conclusions:**

Our findings highlight the considerable productivity losses due to uncorrected and under-corrected presbyopia in LMICs, especially in a longitudinal manner. There is a great need for the development of enabling eye care policies and programs to create access to eye care services, and more healthcare investment in the correction of presbyopia in the working-age population in LMICs. This study could provide evidences for some potential health-related strategies for socio-economic development.

## Highlights

What is already known on this topic

- Tremendous number of people had uncorrected and under-corrected presbyopia in LMICs which would lead to negative impacts on their economic and human's quality of life.
- Global uncorrected and under-corrected presbyopia could result in significant productivity losses. However, productivity loss due to uncorrected and under-corrected presbyopia in LMICs in a longitudinal manner has not been studied.

What this study adds

- In 2019, a total of 54.13 billion (current US dollars) (95% confidence interval [CI]: 34.34-79.02 billion) potential productivity loss was estimated in LMICs.
- In a longitudinal manner, loss of PALYs per presbyopia case was estimated to be 0.7 and total potential productivity loss was predicted to be US$ 315 billion (an average loss of US$ 1453.72 per person).

How this study might affect research, practice or policy

- Considerable productivity losses due to uncorrected and under-corrected presbyopia in both the cross-sectional and longitudinal manner highlight a great need for great need for the development of enabling eye care policies and programs to create access to eye care services, and more healthcare investment in the correction of presbyopia in the working-age population in LMICs.

## Introduction

Presbyopia, resulting from the progressive decline of the eye's accommodation ability, is an age-related impairment of near vision (1). Because of the global growth of life expectancy and the aging society, nowadays the number of people concerned by presbyopia was tremendous and growing rapidly (2). Various reasons such as large population, a high prevalence (ranges from 43.8 to 93.4%), lack of eye health resources and limited access to eye care service, poor awareness of presbyopia, low presbyopia spectacle-correction coverage rates (as low as 10%) and low compliance made low- and middle-income countries (LMICs) suffered more than 90% of the global burden of presbyopia vision impairment (3–6). And it has been estimated that willingness to pay for spectacles was significantly associated with salaries, near visual demand and an independent income source. Compared with people earning <$53.0, people earning more than $107.1 were 48 times more likely to purchase glasses (CI: 8.591–71.758), which explained the higher uncorrected presbyopia prevalence in LMICs in a way (7). Besides, global population aging was considered to be a significant risk factor contributing to presbyopia (8–10). Previous studies showed that 1.8 billion people were affected by presbyopia in 2015, making up 25% of the world population (95% confidence interval [CI], 1.7–2.0 billion). And 826 million people (95% CI, 686–960 million people) of these patients had vision impairment which could lead to difficulties with near vision tasks, because of no, or inadequate, vision correction (1). The number of people with disabilities owing to uncorrected presbyopia was 474.1 million in 2010, of which 448 million were in less- and least-developed countries, accounting for 94.5% of the total (3).

Typically, presbyopia occurs after age 40, and gradually aggravates until age 60. Presbyopia has been proved to lead to negative impacts on human's quality of life and ability to daily activities (11, 12): 53% of Indians (13), 58% of Brazilians (14), and 70% of rural Tanzanians (15) with functional presbyopia had difficulties with near vision tasks, which would further result in significant productivity losses, which includes absence from work and reduced efficiency at work (presenteeism) (16). This effect should be of concern especially given that the increases in retirement age and the employment of older workers have been observed in various countries (17). Therefore, it is important to estimate the productivity losses due to uncorrected presbyopia in LMICs to capture the economic burden of uncorrected presbyopia.

Previous studies have investigated the economic burden of presbyopia in different methods (18–20). Frick et al. estimated that the potential productivity losses due to uncorrected and under-corrected presbyopia could be US$ 25 billion or 0.037% of global GDP in the working-age presbyopia adults (≤65 years) in 2011 (18). Due to the high prevalence of uncorrected and under-corrected presbyopia among people of working age in LMICs, the potential productivity losses could be large. However, the economic burden regarding productivity losses due to presbyopia in LMICs, has not been reported. Moreover, no study has thus far estimated the potential productivity loss due to uncorrected and under-corrected presbyopia in a longitudinal manner.

In the current study, we aimed to quantify the potential productivity loss due to uncorrected and under-corrected presbyopia in LMICs over an extended period of time, measured in terms of productivity-adjusted life years (PALYs) (21–23). PALY is a newly developed measurement akin to quality-adjusted life years, which can adjust years of life lived for productivity losses attributable to chronic health conditions at the population level (21).

## Methods

### The data sources

The prevalence of uncorrected and under-corrected presbyopia in each low- and middle-income country was sourced from the Global Burden of Disease Study (GBD) 2019, stratified by 5-year age groups. Gross domestic product (GDP) represented the sum of value added by all its producers. The data was sourced from the World Bank's World Development Indicator database of 2019 and the newest Central Intelligence Agency's (CIA) World Factbook. The employment-to-population ratio was defined as the proportion of a country's working-age population that was employed. Labor force participation rate was the ratio of the economically active population (including the employed and the unemployed) to the working-age population. We obtained the employment-to-population ratio and Labor force participation rate in 2019 from the International Labor Organization. In general, information for this indicator was derived from household surveys, mainly labor force surveys. More details about data in this article can be found in the Supplementary Tables S1, S2, S3.

### The productivity index and PALY

The PALY, which has been described in previous studies, is a measure of disease burden that can be used to measure the impact of chronic health conditions on work productivity at the population level (21). Briefly, the PALYs are derived from multiplying years of life lived by productivity indices. This is similar to the calculation of quality-adjusted life years (QALYs). The productivity index represents the productivity of an individual in proportional terms, ranging from 1 (100% productive) to 0 (entirely non-productive). The productivity index can be derived from estimates of absenteeism (missed working days), and presenteeism (productivity reduction) in those with uncorrected presbyopia compared with those without uncorrected presbyopia. Absenteeism was defined as the number of lost work days per year due to uncorrected presbyopia, while presenteeism was defined as productivity reduction. The productivity reduction was estimated at 10.9% from previous studies (Supplementary Table S5), while absenteeism was assumed to be 0.

In order to calculate the potential productivity losses caused by uncorrected and under-corrected presbyopia among the working-age people in a longitudinal manner, we introduced the life table models to construct age cohorts (in 5-year age groups) of the working-age population (aged from 15 to 64 years old) in LMICs (24). Firstly, to estimate the burden of presbyopia, the uncorrected and under-corrected presbyopia subjects of the working-age, defined as the “presbyopia cohort”, were followed up in a model simulation over their working lifetime (until the retirement age of 65 years). The same cohort was then re-stimulated but hypothetically assumed to be free of uncorrected and under-corrected presbyopia. This was defined as the “Non-presbyopia cohort”. As uncorrected and under-corrected presbyopia is not a mortality condition, we assumed that no extra deaths in the “presbyopia cohort” compared with the “non-presbyopia cohort”. Thus, in the current study, the differences in the results of the “presbyopia cohort” and “Non-presbyopia cohort” were quantified in terms of the PALYs. The WHO standard 3% annual discount rate was then applied to all years of life and PALYs (25).

### Calculation of the potential productivity loss

To calculate the potential productivity loss due to presbyopia, we multiplied the number of total working-age cases (TC) by the productivity-adjusted life years (PALYs) lost due to presbyopia, employment-to-population ratio (ER), labor participation rate (LPR) and the gross domestic product (GDP) per capital together. The formula used to calculate the total productivity loss (TPL) was TPL = TC ^*^ER^*^LPR^*^PALYs lost^*^ GDP (PC).

### Scenario and sensitivity analysis

Deterministic sensitivity analyses were performed to assess the impact of uncertainty around presbyopia-related productivity indices, and economic data inputs on the model. These included: upper and lower uncertainty bounds of presbyopia cases, upper and lower uncertainty bounds around productivity indices based on decreasing and increasing estimates of presenteeism by 25%. To assess the impact of the assumption of the WHO standard annual discount rate of 3.0%, scenario analyses were performed in which the discount rate was 5.0 and 1.5%, respectively.

## Results

**Figure 1:**
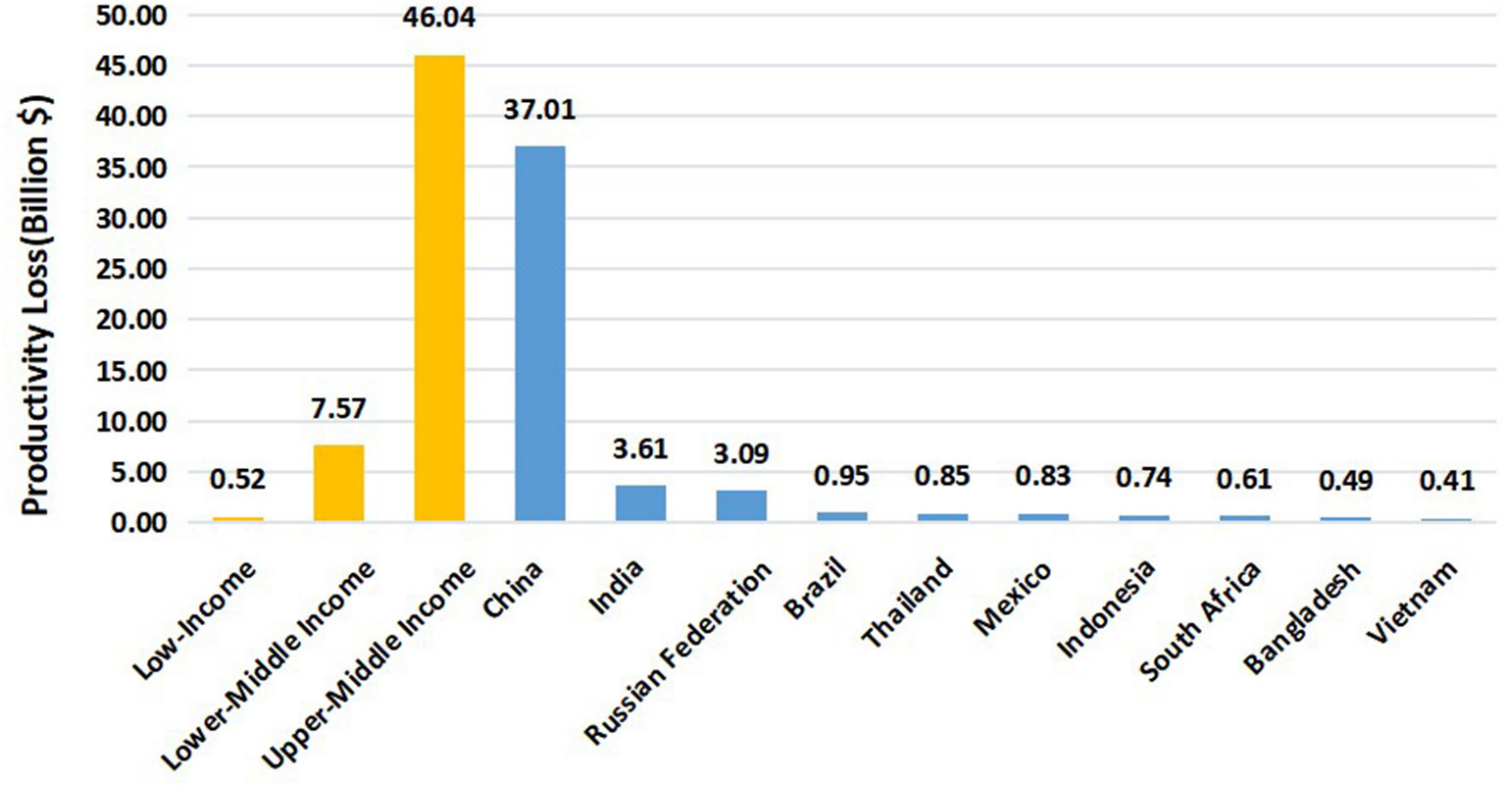
Productivity losses of presbyopia in LMICs and TOP10 countries of 40-64y (billion $) in 2019.

In 2019, the number of global cases of uncorrected and under-corrected presbyopia in working age was 257.07 million, of which 92.7% (238.4 million) were in LMICs (entire data can be found in Supplementary Table S1). Table 1 shows the numbers of presbyopia cases and the productivity losses varying from regions in 5-year age groups (40–64 years old) in 2019. There were 238.40 million (95% confidence interval [CI]: 150.92–346.78 million) people suffering from uncorrected and under-corrected presbyopia in LMICs, which resulted in a productivity loss of 53.41 billion US$ (95% confidence interval [CI]: 34.34–79.02 billion) in 2019. And the percent of productivity losses to GDP in all LMICs is 0.17% (95% CI:0.11–0.25) in 2019. We estimated the productivity losses due to uncorrected and under-corrected presbyopia in every country of LMICs (Supplementary Table S6). The top 10 low- and middle-income countries with the highest productivity loss due to uncorrected and under-corrected presbyopia cases in 2019 are shown in Figure 1. Among all the LMICs, the top 3 countries with the greatest productivity losses were China (37.01 billion, 95% CI 23.63–53.86), India (3.61 billion, 95% CI 2.29–5.29), and Russian Federation (3.09 billion, 95% CI 1.95–4.52), respectively.

**Table 1:** Productivity losses of presbyopia in LMICs in 2019.

**Economies**	**Cases of uncorrected and under-corrected presbyopia by 40–64 y (millions $)**	**Productivity losses of uncorrected and under-corrected presbyopia** **(billions $)**	**GDP (trillions$)**	**Productivity loss/GDP (%)**
Low-Income	12.98 (11.97–17.60)	0.52 (0.33–0.77)	0.49	0.11 (0.07–0.16)
Lower–Middle Income	119.95 (71.10–175.29)	7.57 (4.78–11.09)	7.72	0.10 (0.06–0.14)
Upper–Middle Income	105.47 (67.85–153.89)	46.04 (29.23–67.16)	24.02	0.19 (0.12–0.28)
Total	238.40 (150.92–346.78)	54.13 (34.34–79.02)	32.23	0.17 (0.11–0.25)

Captions: The Economies according to the GBD 2019 study of Low-and Middle-Income Countries: Low-Income Economies ($1,045 OR LESS); Lower–Middle Income Economies ($1,046 TO $4,095); Upper–Middle Income Economies ($4,096 TO $12,695). The number of presbyopia is derived from the GBD 2019 study. The number of cases and productivity losses by presbyopia are given in form of with 95% confidence interval.

**Figure 2:**
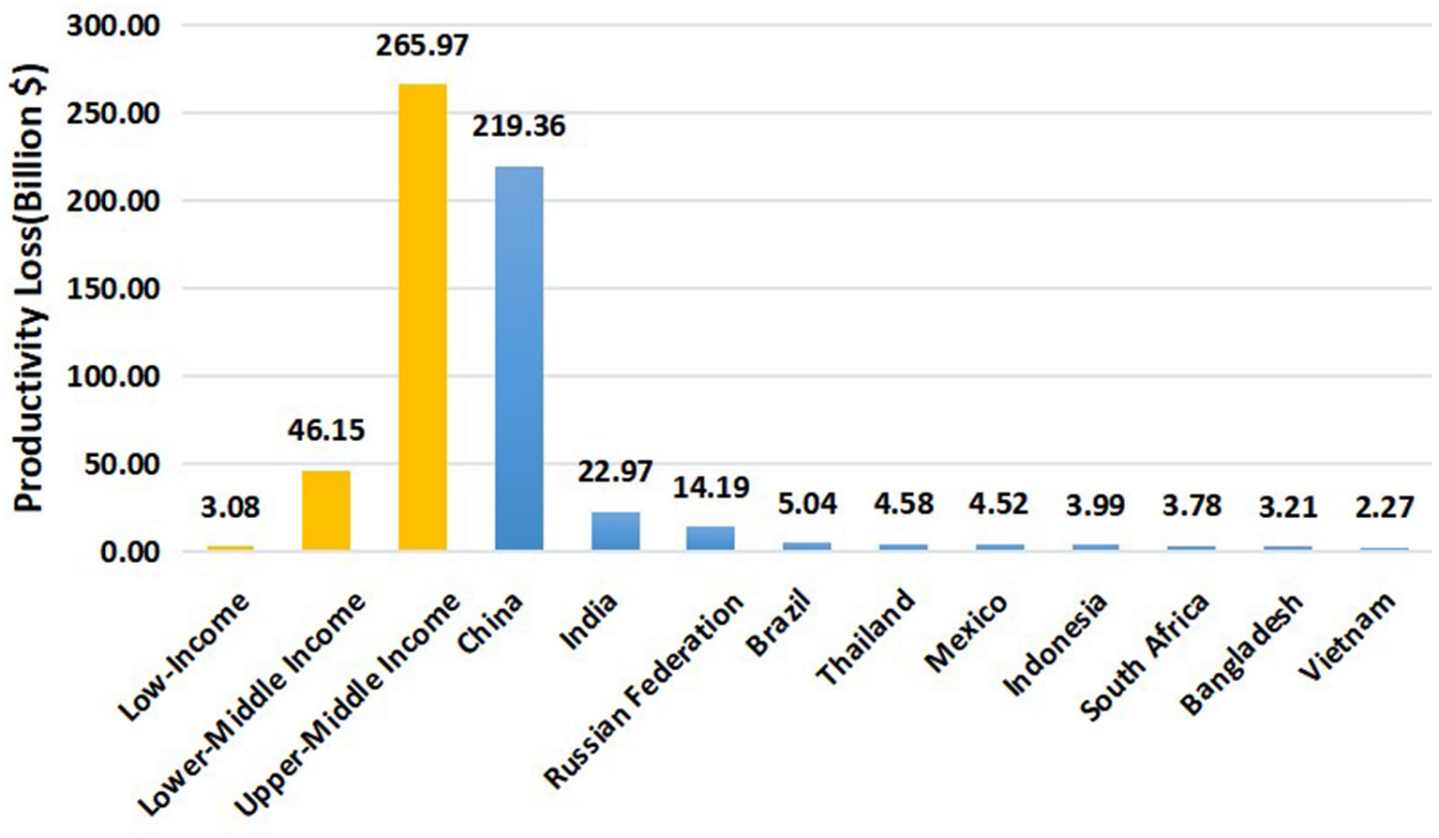
Productivity losses of presbyopia in LMICs and TOP10 countries (from 2019 to retirement age) in billion$.

Table 2 demonstrates that the PALYs lived in those with presbyopia and the same cohort without presbyopia in 5 age groups (40–64 years old). Table 3 and Figure 2 show the productivity losses of presbyopia in LMICs from 2019 to retirement age. With simulated follow-up until retirement (Supplementary Table S4), we estimated that PALYs lived by the current cohort of people with uncorrected and under-corrected presbyopia in LMICs would be reduced by an estimate of 155 million PALYs (10.9%) over their working lifetime, compared with the same cohort assuming no presbyopia. This equated to 0.7 PALYs lost per person with presbyopia over the working lifetime. Based on the gross domestic product (GDP) per full-time worker in 2019, the loss in PALYs equated to a total of 315.20 billion US$ (95%CI 199.24–464.24) in lost GDP owing to reduced productivity, with an average of US$ 1453.72 lost per person with presbyopia. Longitudinally, the top 3 countries with the greatest productivity losses were China (219.36 billion, 95% CI 139.64–322.03), India (22.97 billion, 95% CI 14.54–33.87), and Russian Federation (14.19 billion, 95% CI 8.86–20.99), respectively. The detailed productivity losses of presbyopia in each country from 2019 to retirement age were shown in (Supplementary Table S7).

**Table 2:** Years of life lived in those with presbyopia, and in the same cohort assuming no presbyopia, over the working lifetime of the population simulated from life table modeling.

**Five-year age group**	**Deaths in cohort with presbyopia (million)**	**Deaths in ‘presbyopia cohort' assuming no presbyopia (million)**	**PALYs lived in cohort with presbyopia (million)**	**PALYs lived in ‘presbyopia cohort' assuming no presbyopia (million)**	**PALYs lost per person with presbyopia**
40–44	4.51 (2.90–6.68)	4.51 (2.90–6.68)	257 (165–380)	288 (185–427)	1.7
45–49	6.76 (4.16–10.28)	6.76 (4.16–10.28)	357 (220–544)	401 (247–611)	1.3
50–54	8.02 (5.18–11.54)	8.02 (5.18–11.54)	365 (236–524)	409 (265–589)	1.0
55–59	7.46 (4.59–10.84)	7.46 (4.59–10.84)	235 (145–342)	264 (162–384)	0.5
60–64	4.85 (3.14–6.98)	4.85 (3.14–6.98)	55 (36–80)	62 (40–90)	0.1
Total	31.60 (19.98–46.31)	31.60 (19.98–46.31)	1269 (802–1871)	1425 (900–2100)	0.7

**Table 3:** Productivity losses of presbyopia cases in LMICs from 2019 to retirement age.

**Economies**	**Presbyopia cases in working age (millions $)**	**Productivity losses (billions $)**
Low-Income	10.32 (6.50–15.15)	3.08 (1.92–4.57)
Lower–Middle Income	107.26 (67.94–156.58)	46.15 (29.03–68.22)
Upper–Middle Income	99.25 (62.98–144.73)	265.97 (168.29–391.45)
Total	216.82 (137.42–316.47)	315.20 (199.24–464.24)

Table 4 shows the results of the Scenario and sensitivity analysis. The model was sensitive to a number of inputs such as cases of presbyopia, productivity indices, and the annual discount rate. Compared with the base case, at upper and lower uncertainty bounds of productivity indices estimates, PALYs lost to presbyopia were reduced and increased by 25%, respectively. Increasing the annual discount rate to 5% corresponded to a 9.71% reduction in PALYs lost, and a reduction in the annual discount rate to 1.5% leading to an 8.69% increase in PALYs lost.

**Table 4:** Sensitivity and scenario analyses to assess the impact of the uncertainties around productivity, and economic data inputs on PALYs lost in those with presbyopia in the population and the associated economic impact.

**Analysis**	**PALYs lost**	**% Change in PALYs lost compared with base case**	**GDP lost (US$ billion)**	**GDP lost per person (US$)**
Base	155295828.5		315	1453.72
1. Upper uncertainty bound of presbyopia cases	228879293.3	+ 47.38	464	1466.94
2. Lower uncertainty bound of presbyopia cases	98063705.35	– 36.85	199	1499.89
3. Productivity indices upper uncertainty bound	194119785.7	+ 25.0	394	1817.15
4. Productivity indices lower uncertainty bound	116471871.4	– 25.0	236	1090.29
5. Annual discount rate reduced to 5%	140222212.1	– 9.71	286	1318.97
6. Annual discount rate reduced to 1.5%	168787178.9	+ 8.69	341	1573.25

Sensitivity analysis 1 and 2 apply (1) the upper bound of the 95% CI and (2) the lower bound of the 95% CI around the presbyopia cases, holding all other model inputs constant.

Sensitivity analysis 3 and 4 apply (3) 25% increase and (4) 25% reduction in the productivity reduction rate estimates, holding all other model inputs constant.

Sensitivity analysis 5 and 6 apply an annual discount rate (5) increased to 5% (in line with the WHO standard annual rate) and (6) reduced to 1.5%.

## Discussion

Collectively, our data demonstrate that uncorrected and under-corrected presbyopia created a considerable burden on the economy of LMICs in both the cross-sectional and longitudinal manner. A total of 238.40 million (95% confidence interval [CI] 150.92–346.78 million) presbyopia cases aged from 40 to 64 years old in 2019 were estimated to cause 54.13 billion US$ (current US dollars) (95% confidence interval [CI] 34.34–79.02 billion) reduction of productivity in LMICs. With simulated follow-up until retirement, those with uncorrected and under-corrected presbyopia were predicted to experience an additional loss of 155 million PALYs (an average loss of 0.7 PALYs per person), which was equivalent to a total loss of US$ 315.20 billion (an average loss of US$ 1453.72 per person).

Previous studies have reported the global potential productivity loss due to distant and near visual impairment (19, 26–28). By applying the near vision impairment disability weight (0.013), Frick et al. estimated that the potential productivity loss due to uncorrected and under-corrected presbyopia could be US$ 25 billion in the working-age presbyopia adults (≤65 years) in 2011 (18). In the current study, by using data from the GBD 2019, we estimated significant greater productivity loss compared with Frick's study, even in LMICs. One possible explanation is that we used the 10.9% of productivity reduction instead of the disability weight (1.3%). We believe that productivity loss calculation using disability weight has limitations. Firstly, disagreement exists regarding the disability weight in the literature (18, 29, 30). Secondly, disability weight may not represent a reduction of productivity. The relative increase of productivity was reported to range from 6 to 21.7%, which is greatly higher than the disability weight. For example, in a recent randomized controlled, investigator-masked trial, Reddy et al. showed that a 21.7% relative productivity increase could be made if presbyopia tea workers were corrected with spectacles in India (a typical country from the LMICs) (19).

The potential productivity loss due to uncorrected and under-corrected presbyopia in a longitudinal manner has not been studied previously. In the current study, we introduced the PALY metric and used the multistage life table models to quantify the future productivity losses among the working-age population. We estimated that uncorrected or under-corrected presbyopia leads to the loss of 0.7 PALYs per case in our simulation. And those with uncorrected and under-corrected presbyopia in LMICs were predicted to cause a total loss of US$ 315 billion (an average loss of US$ 1453.72 per person) in our study. Although the burden of uncorrected presbyopia was also indispensable in high-income countries, people in these regions seem to have more opportunities to get the adequate optical correction (31). And Frick et al. found that the productivity loss of LMICs was much higher than high-income countries in 2011. The reasons for this difference might be complicated, including variations in wages, productivity, age structures, and employment rates (18). The poor are less able to afford spectacles for correction of presbyopia, so there is a great need for the development of enabling eye care policies and programs to create access to eye care services, and more healthcare investment in the correction of presbyopia in the working-age population in LMICs. It's worth to concern that a majority of the productivity loss by presbyopia in LMICs were estimated in the top 10 (89.77%), especially the top 3 (80.75%) countries (Figures 1, 2). Interventions to diagnose presbyopia (alongside other potential eye conditions) and increase spectacle coverage are highly recommended in these countries. Meanwhile, screening of presbyopia is beneficial to develop integrated eye care systems, because people with presbyopia are more likely to have sight-threatening diseases such as cataracts, glaucoma, and diabetic eye disease (6).

Clinically, there are several strategies to correct presbyopia (32). Correction with spectacles is known as the most common way that is simplest and cost-effective. Forms of spectacle lens include readers, single-vision glasses, bifocals, trifocals and progressive which are relatively mature now (33, 34). Single-vision or progressive contact lenses could also be used in presbyopia correction (35). Surgical approaches were considered to correct presbyopia too. But this way might be expensive and intractable which is an invasive procedure (35–40). Other options such as pharmacological therapies and ciliary muscle electrostimulation have not been widely applied in clinics yet (41, 42). Generally, obtaining spectacles is vital for the uncorrected presbyopia population, which was reported to be relative low cost (minimum price of a pair of spectacles: 8.6 to 12.9 US $) in previous studies (7, 43, 44). And a study in 2020 indicated that compared with the standard international cost of spectacles in 2006 (US $ 3.0) (45), the cost of which presbyopia people willing to pay for a pair of spectacles (US $ 19.0) was much higher. Moreover, the under-49 age group was 6.2 times more likely to get a pair of spectacles than the over-60 age group (7). Besides, a study in India suggested that most barriers to presbyopia correction were lack of ‘felt need' (46%), ‘lack of awareness' of presbyopia symptoms (16%), ‘lack of access' (13%) and economic (13%) and personal reasons (12%)(46). So popularization of basic medical knowledge about presbyopia also plays an important role in the correction of presbyopia. We hope that the heavy productivity loss of the presbyopia population can attract the attention of the state, so as to expand the popularization of presbyopia and its correction. Additionally, we hope this study would inspire further related studies, such as near vision loss and socio-economic influence in LMICs, cost-effective strategies for presbyopia correction, and provision of eye care. And the ultimate goal is to improve the visual acuity and quality of life of presbyopia people through simple and effective means, of course, to a certain extent, to improve the economic level of the country.

This study has some limitations. Firstly, there were only 4 previous studies reporting the relative productivity reduction associated with presbyopia (Supplementary Table S5), and there were weaknesses in the GBD data source and methodology, including limited quality of data, and sparsity of population-based data, which could result in bias. Therefore, more robust data on presbyopia associated productivity reduction, especially from LMICs, is needed to improve our estimates. Secondly, the well-known limitation “life table assumption” in life table modeling should be noted, which means that age-specific mortality did not change over time in our modeling (46). Fortunately, presbyopia is non-fatal and the assumption was applied in both presbyopia and normal population, so the estimates would not change significantly. Thirdly, the productivity index applied in this study may not be generalizable to all LMICs, since not all economically productive activities were influenced by presbyopia in same degree. Many economically important activities, such as cooking, writing, reading, sewing, weeding, use of cell phones, and recognizing money, would be assigned with various weights in our further studies. Fourthly, this study only accounted the indirect costs of presbyopia in employment works, without considering the difficulties with near-vision tasks in unemployment work, like reading, writing, and using mobile phones, which were also important skills in production. Also, we did not take into account the onset of patients in the future (21), which could result in underestimate in productivity losses.

In conclusion, our findings highlight considerable productivity losses due to uncorrected and under-corrected presbyopia in both the cross-sectional and longitudinal manner in LMICs. There is a great need for the development of enabling eye care policies and programs to create access to eye care services, and more healthcare investment in the correction of presbyopia in the working-age population in LMICs. This study could provide evidences for some potential health-related strategies for socio-economic development.

## Synopsis/precis

We found that uncorrected and under-corrected presbyopia in LMICs were predicted to cause a total loss of US$ 315 billion (an average loss of US$ 1453.72 per person) and a loss of 0.7 PALYs per case in low- and middle-income countries (LMICs).

## Data availability statement

Publicly available datasets were analyzed in this study. This data can be found here: https://www.healthdata.org/gbd/2019, https://databank.worldbank.org/source/world-development-indicators, https://www.cia.gov/the-world-factbook/. The other raw data supporting the conclusions of this article can be made available by the authors, without undue reservation.

## Author contributions

Study design and manuscript writing and review: KQ and ZG. Data collection and analysis: QM, MC, and DL. Data analysis and interpretation: RZ, YD, SY, BC, HW, and JJ. All authors contributed to the article and approved the submitted version.

## Funding

This study was partly supported by Special Fund for Science and Technology of Guangdong Province (Grant No. 2019ST024) and the Science and Technology Plan Project of Shantou (Grant No. 汕府科 [2019]106).

## Conflict of interest

The authors declare that the research was conducted in the absence of any commercial or financial relationships that could be construed as a potential conflict of interest.

## Publisher's note

All claims expressed in this article are solely those of the authors and do not necessarily represent those of their affiliated organizations, or those of the publisher, the editors and the reviewers. Any product that may be evaluated in this article, or claim that may be made by its manufacturer, is not guaranteed or endorsed by the publisher.
