# Supplementary material for: Potential productivity loss from uncorrected and under-corrected presbyopia in low- and middle-income countries: A life table modeling study
Source: Front Public Health. 2022 Oct 11;10:983423. doi: 10.3389/fpubh.2022.983423 (PMC9592832; doi:10.3389/fpubh.2022.983423)
Supplement: Supplementary file 1 [file Table_1.DOCX]

**Supplementary Materials**

**The file list of the Supplementary Materials:**

| **Supplementary Table S1** | **Number of Presbyopia cases in LMICs in 2019.** |
| --- | --- |
| **Supplementary Table S2** | **ER、LPR、Per Capital GDP、GDP and population (40-64 years old) in LMICs in 2019.** |
| **Supplementary Table S3** | **Mortality in 2019 by age group.** |
| **Supplementary Table S4** | **Number of presbyopia cases followed up to retirement by age group.** |
| **Supplementary Table S5** | **Work productivity gaps due to vision loss (presbyopia) identified in the literature.** |
| **Supplementary Table S6** | **Productivity loss of Presbyopia in 2019 by age group in Millions US$.** |
| **Supplementary Table S7** | **Productivity loss of Presbyopia in 2019 by age group until retirement in Millions US$.** |

**Notes:**

1. **NO DATA of cases of presbyopia(2 countries)：West Bank and Gaza/Kosovo.**
2. **NO DATA of LPR(6 countries)：Micronesia, Fed.Sts./American Samoa /Dominica /Grenada / Marshall Islands/Tuvalu.**

**Table S1. Number of Presbyopia cases in LMICs in 2019.**

| **Location** | | **40-44Y** | | **45-49Y** | **50-54Y** | | **55-59Y** | | | | **60-64Y** | **40-64Y** |
| --- | --- | --- | --- | --- | --- | --- | --- | --- | --- | --- | --- | --- |
| China | | 5008165  (3244217-7413287) | | 11306536  (7030641-17231392) | 18651654  (12159267-26637285) | | 20710532  (12842091-30149016) | | | | 21595280  (14055744-31027659) | 77272167  (49331960-112458641) |
| India | | 9314221  (6049297-13704413) | | 13891443  (8604564-21064015) | 16411951  (10574451-23820674) | | 17603287  (10762289-25487922) | | | | 17190481  (11105196-24846733) | 74411383  (47095797-108923757) |
| Bangladesh | | 947000  (603287-1406228) | | 1422352  (844827-2205221) | 1717365  (1076251-2532299) | | 1872537  (1129046-2747886) | | | | 1773950  (1115791-2630022) | 7733204  (4769202-11521656) |
| Russian Federation | | 404057  (253925-611398) | | 703142  (424571-1073855) | 1095843  (710346-1567486) | | 2056004  (1253988-3036411) | | | | 2691605  (1739267-3892236) | 6950650  (4382097-10181386) |
| Pakistan | | 671648  (433394-984647) | | 1003780  (626333-1504619) | 1185920  (778134-1665474) | | 1240333  (810526-1683036) | | | | 1129485  (775030-1510775) | 5231166  (3423418-7348552) |
| Nigeria | | 595491  (378374-878394) | | 919090  (563771-1404698) | 1189642  (767944-1697114) | | 1321880  (813432-1939252) | | | | 1187510  (771230-1707821) | 5213615  (3294750-7627279) |
| South Africa | | 541912  (369837-768476) | | 782340  (501586-1152262) | 907917  (610031-1278987) | | 981400  (629106-1377428) | | | | 924167  (627389-1274083) | 4137735  (2737949-5851236) |
| Indonesia | | 316059  (191698-486310) | | 512777  (299172-794296) | 778373  (484048-1136029) | | 1021234  (611559-1531016) | | | | 1144447  (719500-1670555) | 3772890  (2305976-5618206) |
| Nepal | | 401894  (294982-511537) | | 656758  (463507-883447) | 732415  (550527-935005) | | 684959  (501466-853410) | | | | 563888  (413227-699768) | 3039913  (2223709-3883166) |
| Brazil | | 219335  (132328-337522) | | 346535  (201935-542342) | 572650  (349980-829118) | | 817475  (477488-1229795) | | | | 1010634  (629908-1456780) | 2966630  (1791640-4395557) |
| Tanzania | | 402303  (253710-640317) | | 629335  (391126-974952) | 675447  (465257-915902) | | 606400  (433573-777465) | | | | 488655  (365900-626940) | 2802139  (1909566-3935576) |
| Vietnam | | 189396  (113866-292413) | | 324758  (188840-519565) | 498200  (321232-727928) | | 692564  (427337-1036862) | | | | 745993  (475987-1101972) | 2450911  (1527262-3678740) |
| Mexico | | 174872  (107414-268474) | | 300351  (178014-463307) | 464192  (288471-671080) | | 623969  (366004-932865) | | | | 753750  (473208-1093228) | 2317134  (1413111-3428953) |
| Thailand | | 144800  (90666-222504) | | 272141  (159841-424218) | 459040  (280713-667138) | | 649892  (377881-963482) | | | | 766214  (488215-1106165) | 2292086  (1397316-3383507) |
| Congo, Dem. Rep | | 187238  (116136-285031) | | 275978  (163101-425073) | 364239  (230399-535693) | | 424285  (257453-637762) | | | | 401446  (259027-589597) | 1653185  (1026115-2473156) |
| Ukraine | | 83621  (50641-130575) | | 147563  (85666-227142) | 245470  (152847-353663) | | 438965  (259328-657529) | | | | 601563  (382886-879615) | 1517183  (931369-2248523) |
| Philippines | | 108982  (65914-169475) | | 175817  (102469-270644) | 269955  (166080-388549) | | 356304  (207063-532221) | | | | 421318  (262580-613217) | 1332376  (804105-1974106) |
| Ethiopia | | 159771  (99315-241959) | | 215193  (128630-327506) | 272547  (175122-391474) | | 319901  (196179-471161) | | | | 343867  (220023-494859) | 1311278  (819269-1926959) |
| Korea, Dem. People's Rep | | 88461  (55178-134033) | | 193206  (115865-301081) | 306738  (193493-453954) | | 338229  (205278-510706) | | | | 344701  (214746-504290) | 1271335  (784561-1904064) |
| Myanmar | | 99979  (61877-152105) | | 166117  (97233-262151) | 252407  (157701-365132) | | 333102  (204145-495021) | | | | 363390  (232435-521616) | 1214994  (753392-1796026) |
| Colombia | | 80993  (48447-122199) | | 140001  (82447-215917) | 238970  (148699-345612) | | 336448  (197679-504844) | | | | 385780  (245613-560780) | 1182194  (722886-1749352) |
| Turkey | | 91911  (55785-144086) | | 137381  (79465-216204) | 215789  (132022-315721) | | 303066  (175513-454981) | | | | 359398  (228389-523772) | 1107545  (671174-1654762) |
| Egypt, Arab Rep. | | 86402  (50649-132904) | | 127119  (72592-196822) | 193887  (117906-284588) | | 256239  (145446-388728) | | | | 280305  (171512-417091) | 943951  (558105-1420132) |
| Niger | | 136385  (101506-182798) | | 185118  (132717-249147) | 198524  (147435-256122) | | 179804  (131830-230433) | | | | 134940  (100997-172703) | 834770  (614485-1091203) |
| Kenya | | 83108  (52230-126462) | | 121432  (73038-186572) | 161806  (103348-232400) | | 197599  (121187-293941) | | | | 204383  (131769-296818) | 768328  (481572-1136193) |
| Uzbekistan | | 62229  (38419-93866) | | 99670  (59019-152862) | 155285  (98874-223632) | | 222962  (134070-330063) | | | | 217095  (139280-318956) | 757241  (469663-1119378) |
| Uganda | | 94141  (57564-145164) | | 135811  (80295-210347) | 165940  (105430-240818) | | 185171  (111836-271182) | | | | 168236  (108797-244519) | 749298  (463923-1112030) |
| Iran, Islamic Rep | | 62703  (36795-98434) | | 92555  (52647-144378) | 140373  (83816-206132) | | 180868  (102990-274379) | | | | 229782  (140839-333809) | 706281  (417086-1057132) |
| Ghana | | 90989  (56277-139593) | | 129345  (78076-198728) | 154003  (99306-221066) | | 168072  (105664-243020) | | | | 162604  (106122-229182) | 705014  (445446-1031589) |
| Romania | | 45055  (27518-69332) | | 79686  (46002-124497) | 129776  (83938-190030) | | 157216  (96725-234859) | | | | 277923  (179672-403366) | 689657  (433855-1022084) |
| Zimbabwe | | 109229  (69667-162045) | | 138668  (84192-215500) | 139473  (88864-204094) | | 141158  (84884-204637) | | | | 135696  (87956-194674) | 664223  (415562-980951) |
| Madagascar | | 78062  (47847-115616) | | 114128  (67641-174521) | 141753  (89973-205233) | | 158106  (97294-233871) | | | | 144221  (93177-208662) | 636270  (395932-937903) |
| Cameroon | | 84114  (54036-128008) | | 114323  (68373-174294) | 132288  (84394-191536) | | 141931  (86254-208117) | | | | 126465  (81461-183443) | 599123  (374519-885397) |
| Malaysia | | 49271  (29317-74834) | | 76951  (44849-119393) | 118706  (74926-172994) | | 163570  (95153-244800) | | | | 189209  (117088-276326) | 597707  (361334-888347) |
| Peru | | 50594  (30523-77734) | | 80831  (47209-127924) | 121586  (75968-178027) | | 159333  (94164-241932) | | | | 182213  (115481-270021) | 594558  (363344-895638) |
| Côte d'Ivoire | | 88174  (55797-134118) | | 117563  (69822-182713) | 131781  (81745-192231) | | 137365  (83411-203785) | | | | 117970  (75441-175115) | 592853  (366216-887960) |
| Mozambique | | 72492  (45449-107996) | | 106432  (64787-164687) | 126228  (80171-183819) | | 138727  (85046-204929) | | | | 135631  (87208-196756) | 579510  (362661-858188) |
| Sri Lanka | | 38838  (23753-60404) | | 65356  (38256-103353) | 107388  (67173-157549) | | 157714  (94887-239702) | | | | 192642  (123105-281923) | 561937  (347174-842931) |
| Belarus | | 29523  (18437-44066) | | 53095  (31705-80417) | 89377  (56312-129850) | | 157670  (94332-239428) | | | | 198077  (128169-290475) | 527742  (328955-784237) |
| Angola | | 63038  (39424-94777) | | 86992  (52424-132772) | 114406  (72921-167346) | | 133472  (79580-203266) | | | | 120511  (77675-179096) | 518419  (322022-777257) |
| Burkina Faso | | 71350  (45394-105854) | | 98670  (60408-149340) | 115489  (74054-166068) | | 122830  (77218-179868) | | | | 108937  (70602-156111) | 517276  (327677-757240) |
| Morocco | | 38920  (22826-62818) | | 61926  (34933-98608) | 95639  (58256-143229) | | 136365  (78789-206964) | | | | 156541  (97719-229431) | 489392  (292524-741050) |
| Kazakhstan | | 35953  (21875-54767) | | 60499  (35387-93067) | 93327  (57555-136048) | | 141805  (84293-210325) | | | | 155255  (98138-225603) | 486839  (297248-719809) |
| Algeria | | 42889  (25544-66184) | | 66226  (37738-102022) | 98215  (60738-143089) | | 125568  (72891-190303) | | | | 144127  (91421-213245) | 477025  (288331-714844) |
| Mali | | 60694  (38654-89817) | | 87332  (53186-131661) | 105201  (67015-152310) | | 114090  (69451-167888) | | | | 104798  (67458-152957) | 472115  (295764-694633) |
| Somalia | | 70223  (44450-102656) | | 85100  (51694-130919) | 73446  (46820-106037) | | 88207  (54197-129638) | | | | 98193  (64040-140614) | 415170  (261202-609863) |
| Senegal | | 46652  (29903-70336) | | 66866  (40694-104152) | 82627  (51295-121578) | | 94043  (57835-139008) | | | | 87001  (54978-124677) | 377190  (234705-559752) |
| Cuba | | 21150  (12845-31972) | | 51828  (29810-79618) | 87233  (55836-126325) | | 112263  (69383-167162) | | | | 100977  (65809-147344) | 373452  (233684-552420) |
| Malawi | 48989(31469-73249) | | 67916(40749-101800) | | 75461(48450-108562) | | 83247(50845-124278) | | 88235(56506-130089) | | | 363848(228019-537978) |
| Iraq | | 34699(20818-53663) | | 56391(32418-87947) | 71680(44481-106457) | | 84662(50880-128369) | | | | 92707(59188-135415) | 340138(207786-511852) |
| Zambia | | 47351(29860-71919) | 64853(38889-98988) | | 74299(46899-108181) | | 77501(44917-115360) | | | | 71785(45078-105303) | 335789(205644-499751) |
| Cambodia | | 26305(15992-40340) | | 41589(24971-63931) | 69023(44416-101535) | | 91251(55831-139048) | | | | 96593(62214-142383) | 324760(203424-487237) |
| Rwanda | | 37479(23674-55540) | | 50477(30003-76967) | 66078(42193-95857) | | 82321(49532-124273) | | 83559(53074-124468) | | | 319915(198476-477106) |
| Azerbaijan | | 19946(12248-30283) | | 35168(20725-54852) | 64029(40036-92802) | | 99052(59748-146614) | | | | 99934(64610-144788) | 318128(197367-469340) |
| Chad | | 43293(27216-65779) | | 61439(37217-96366) | 69857(44039-103749) | | | 72183(45035-104110) | | | 63223(40605-91086) | 309995(194112-461091) |
| Serbia | | 17736(10731-27259) | | 30555(17757-47061) | 50414(31550-72357) | | 76523(45749-113855) | | 120822(77656-177030) | | | 296050(183443-437562) |
| Sudan | | 32432(19777-49694) | | 47353(28064-74473) | | 63781(39479-93135) | 71443(42193-106876) | | | | 76935(47618-114082) | 291945(177131-438260) |
| Ecuador | | 23624(14072-36033) | | 37432(22142-58386) | 55468(33626-80938) | | 76295(44519-114999) | | | | 85613(52603-123002) | 278433(166962-413357) |
| Guinea | | 35751(22743-53775) | | 50954(30940-77211) | 59352(37929-86168) | | 64666(39287-94992) | | | | 61714(39478-90947) | 272437(170377-403093) |
| Burundi | | 32350(20486-48926) | | 44087(26528-66378) | 57045(36182-81953) | | 67234(41623-98083) | | | | 64336(41360-94508) | 265052(166180-389847) |
| Guatemala | | 26523(16470-42272) | | 37317(22156-59622) | 51364(32354-74535) | | 67928(40512-100835) | | | | 78879(50411-114891) | 262010(161903-392155) |
| Benin | | 36897(23393-54669) | | 51109(31639-77416) | 57543(36584-83823) | | 59203(36448-86941) | | | | 52778(33906-76657) | 257529(161971-379505) |
| Bulgaria | | 15391(9285-23671) | | 27655(16305-42506) | 44012(27588-64492) | | 68047(40526-100510) | | | | 96210(61224-138804) | 251316(154928-369982) |
| Afghanistan | | 24641(14398-38025) | | 54144(30764-83664) | 63790(39115-93619) | | 46119(27292-69952) | | | | 35887(22913-53092) | 224580(134482-338351) |
| South Sudan | | 27375(17204-41045) | | 41503(24852-64546) | 51846(32864-75252) | | 53463(33191-79184) | | | | 47650(30730-70529) | 221837(138841-330556) |
| Dominican Republic | | 21041(13047-31994) | | 33178(19618-51014) | 46311(29220-67892) | | 56727(34149-86126) | | | | 59016(37458-86559) | 216272(133493-323585) |
| Yemen, Rep. | | 25997(15455-40311) | | 32571(18675-50525) | 42366(25624-61922) | | 53324(31396-80299) | | | | 60653(37821-89427) | 214911(128971-322483) |
| Moldova | | 12730(7558-19077) | | 21108(12455-32328) | 33868(21246-49166) | | 59320(35569-88913) | | | 72093(45298-105742) | | 199118(122126-295225) |
| Togo | | 27509(17644-40971) | | 39393(24205-59753) | 45583(28906-65448) | | 47040(27977-68867) | | | | 39280(25017-56767) | 198805(123749-291806) |
| Haiti | | 22994(13857-34571) | | 31695(18574-48116) | 40589(25773-58424) | | 48567(29477-71657) | | | | 50171(31518-73567) | 194016(119199-286334) |
| Tajikistan | | 15519(9387-24103) | | 25678(14986-40633) | 40868(25880-59451) | | 56639(34654-85142) | | | | 54066(35161-79557) | 192770(120069-288886) |
| Syrian Arab Republic | | 15088(8982-23250) | | 26061(14993-40703) | 41011(25527-59767) | | 52979(32080-78882) | | | | 55767(35775-80341) | 190905(117356-282943) |
| Sierra Leone | | 26436(16773-39102) | | 36225(21593-55544) | 41356(26214-60147) | | 42970(25943-62487) | | | | 38539(24296-55835) | 185527(114819-273115) |
| Bolivia | | 16242(10005-24924) | | 24499(14372-38213) | 35152(22237-51441) | | 46720(27300-70746) | | | | 54581(34446-79897) | 177194(108360-265221) |
| Tunisia | | 12985(7725-20651) | | 19685(11418-31418) | 31923(20046-47139) | | 46472(26828-70167) | | | | 55913(34261-82638) | 166978(100278-252014) |
| Eritrea | | 22904(15021-34145) | | 33304(20618-50620) | 37640(24312-54209) | | 37757(23392-54619) | | | | 32240(20162-46565) | 163845(103504-240157) |
| Honduras | | 15220(9399-23327) | | 22807(13208-35179) | 31218(19318-45389) | | 40216(23767-60406) | | | | 44981(29063-67107) | 154442(94754-231409) |
| Kyrgyz Republic | | 11441(7016-16897) | | 19528(11608-29977) | 30441(18840-44748) | | 43651(26002-66409) | | | | 44975(28491-65902) | 150035(91958-223934) |
| Papua New Guinea | | 16191(9789-24841) | | 24382(14333-38073) | 32960(20788-48062) | | 38067(22100-57102) | | | | 36704(22951-53439) | 148305(89961-221517) |
| Bosnia and Herzegovina | | 7385(4414-11297) | | 12842(7585-19890) | 23331(14670-33848) | | 38563(22242-57423) | | | | 51722(32271-76291) | 133844(81182-198749) |
| Botswana | | 20186(12964-30004) | | 25859(16091-40058) | 27245(17398-39739) | | 29366(17882-42830) | | | | 26256(16780-37898) | 128912(81114-190530) |
| Georgia | | 7074(4222-11190) | | 12627(7487-19928) | 21608(13321-31819) | | 37689(21747-56748) | | | | 49268(30676-71427) | 128266(77452-191112) |
| El Salvador | | 10499(6537-16024) | | 16877(10083-26066) | 25465(15939-37161) | | 33702(20707-51082) | | | | 39369(25111-57901) | 125912(78376-188234) |
| Lao PDR | | 11625(7107-17988) | | 18128(10634-27744) | 26006(16141-38134) | | 32870(19161-48868) | | | | 33719(21447-49460) | 122347(74490-182195) |
| Namibia | | 17370(11241-25620) | | 24318(14847-37365) | 27126(17624-39886) | | 27512(17214-40406) | | | | 24529(15800-35244) | 120855(76726-178521) |
| Turkmenistan | | 9304(5708-14179) | | 15450(9064-23909) | 24075(15405-34347) | | 34841(21186-52232) | | | | 35632(22656-51637) | 119303(74020-176303) |
| Nicaragua | | 10903(6790-17373) | | 16627(9872-26495) | 24199(15236-35090) | | 31321(18673-46469) | | | | 34420(21995-50147) | 117471(72567-175574) |
| Liberia | | 18387(11702-27536) | | 27057(16417-41721) | 24628(15680-35567) | | 25064(15106-36779) | | | | 21189(13322-30637) | 116324(72226-172240) |
| Costa Rica | | 8197(4924-12491) | | 13333(7897-20332) | 22955(14548-33146) | | 33165(20128-49804) | | | | 38156(24873-56173) | 115806(72370-171947) |
| Congo, Rep. | | 14005(8508-21451) | | 20414(11854-31736) | 24618(15588-35378) | | 26697(16279-39893) | | | | 24629(15814-35885) | 110363(68042-164345) |
| Paraguay | | 9144(5427-14269) | | 13938(8045-22021) | 21162(12937-30245) | | 29094(17400-43552) | | | | 33026(20737-47638) | 106365(64546-157724) |
| Lesotho | | 15012(9868-22039) | | 18581(11473-28268) | 22802(14735-33095) | | 25504(16016-37062) | | | | 23241(15176-33325) | 105140(67268-153788) |
| Armenia | | 5836(3577-8890) | | 9337(5427-14518) | 16396(10236-23801) | | 31306(18658-48130) | | | | 41417(25738-61039) | 104292(63635-156379) |
| Central African Republic | | 12552(7792-18780) | | 18506(11326-28260) | 23681(14526-34200) | | 25653(14955-38583) | | | | 23747(14666-35004) | 104138(63266-154827) |
| Albania | | 4729(2891-7353) | | 9679(5844-15118) | 18358(11585-26626) | | 30498(17492-45621) | | | | 38915(23969-56893) | 102178(61781-151612) |
| Jordan | | 10114(5925-15728) | | 16200(9230-25380) | 23110(14027-34103) | | 26702(15221-41512) | | | | 25918(15988-38367) | 102045(60392-155090) |
| Argentina | | 9092(4880-15017) | | 12881(6678-21102) | 17994(9925-27829) | | 25254(13009-40214) | | | | 33040(18881-52393) | 98259(53373-156555) |
| Mauritania | | 11954(7531-17712) | | 17518(10593-26251) | 21182(13130-30301) | | 23615(14037-34362) | | | | 22665(14128-33487) | 96934(59419-142112) |
| Panama | | 6774(4064-10237) | | 11802(6869-18384) | 18249(11580-26698) | | 24201(14591-36032) | | | | 27532(17616-39922) | 88559(54719-131272) |
| Mongolia | | 7318(4491-11228) | | 11696(6934-18125) | 17408(10994-24945) | | 22362(13290-33583) | | | | 20467(13132-29753) | 79251(48842-117634) |
| North Macedonia | | 4654(2887-7077) | | 8290(4961-12723) | 14244(9117-20567) | | 21285(13006-31306) | | | | 27733(18078-40816) | 76206(48049-112488) |
| Libya | | 8503(5013-13228) | | 12772(7409-20098) | 16573(10339-24106) | | 18096(10507-27173) | | | | 18211(11321-26507) | 74153(44590-111112) |
| Jamaica | | 5019(3085-7756) | | 8805(5204-13570) | 13106(8314-19098) | | 17016(10382-25277) | | | | 18459(12059-27281) | 62405(39043-92982) |
| Lebanon | | 4703(2830-7305) | | 7849(4530-12217) | 11576(7260-16974) | | 16262(9990-24731) | | | | 17888(11406-26345) | 58278(36016-87572) |
| Eswatini | | 7702(4956-11396) | | 9928(5992-15162) | 10898(6905-15780) | | 11218(6863-16676) | | | | 10512(6779-15445) | 50257(31496-74459) |
| Gambia, The | | 6798(4304-10183) | | 8935(5363-13952) | 10113(6447-14851) | | 10410(6410-15238) | | | | 9810(6229-14173) | 46066(28752-68398) |
| Gabon | | 4205(2606-6458) | | 6487(3879-9922) | 8754(5505-12565) | | 10525(6337-15563) | | | | 10180(6486-14727) | 40150(24813-59235) |
| Guinea-Bissau | | 5688(3619-8535) | | 7541(4506-11451) | 8582(5519-12422) | | 8889(5548-13250) | | | | 7892(5088-11549) | 38592(24280-57207) |
| Mauritius | | 2444(1500-3746) | | 3975(2323-6206) | 7396(4569-10917) | | 11290(6780-17079) | | | | 13170(8349-19409) | 38275(23522-57358) |
| Bhutan | | 4578(2914-6843) | | 6475(3925-9912) | 7641(4798-11169) | | 8240(4867-12151) | | | | 7743(4790-11376) | 34677(21293-51450) |
| Djibouti | | 4599(2867-6908) | | 6463(3833-9940) | 7629(4886-11129) | | 8059(4838-11972) | | | | 7380(4617-10662) | 34130(21041-50612) |
| Comoros | | 2503(1591-3774) | | 3952(2370-6084) | 5084(3233-7276) | | 5675(3430-8386) | | | | 5524(3566-8078) | 22738(14189-33598) |
| Montenegro | | 1256(760-1922) | | 2191(1252-3418) | 3769(2359-5558) | | 6046(3731-9000) | | | | 8081(5149-11502) | 21342(13252-31400) |
| Cabo Verde | | 2047(1282-3032) | | 3096(1836-4841) | 4285(2695-6243) | | 5055(3122-7323) | | | | 4867(3115-7103) | 19351(12051-28541) |
| Timor-Leste | | 1698(1030-2570) | | 2922(1732-4579) | 3685(2310-5427) | | 4388(2515-6603) | | | | 6417(3974-9434) | 19111(11561-28613) |
| Fiji | | 1492(903-2307) | | 2366(1394-3652) | 3894(2384-5737) | | 5429(3150-8289) | | | | 5569(3494-8204) | 18750(11323-28188) |
| Equatorial Guinea | | 2364(1502-3525) | | 3255(1936-5093) | 4028(2478-5937) | | 4428(2566-6725) | | | | 4321(2708-6355) | 18396(11189-27634) |
| Guyana | | 1434(879-2222) | | 2323(1337-3702) | 3346(2073-4966) | | 4269(2498-6432) | | | | 4362(2734-6473) | 15735(9521-23795) |
| Suriname | | 1097(680-1699) | | 1989(1184-3026) | 3177(1997-4679) | | 4017(2391-5969) | | | | 3980(2508-5884) | 14260(8760-21259) |
| Maldives | | 1039(632-1612) | | 1416(810-2229) | 1859(1134-2706) | | 2268(1285-3397) | | | | 2268(1432-3255) | 8850(5292-13200) |
| Solomon Islands | | 1054(630-1623) | | 1555(896-2408) | 1903(1186-2775) | | 2144(1270-3181) | | | | 2069(1316-3010) | 8725(5298-12997) |
| Belize | | 739(455-1117) | | 1158(691-1778) | 1538(983-2226) | | 1876(1126-2760) | | | | 1862(1160-2713) | 7173(4415-10595) |
| São Tomé and Principe | | 724(463-1068) | | 1040(626-1576) | 1188(753-1731) | | 1293(786-1925) | | | | 1132(711-1667) | 5377(3339-7968) |
| St. Lucia | | 389(235-595) | | 701(403-1084) | 1076(667-1558) | | 1325(794-1961) | | | | 1363(861-2018) | 4853(2959-7217) |
| Vanuatu | | 422(257-655) | | 647(378-988) | 889(551-1308) | | 1097(646-1645) | | | | 1283(805-1872) | 4338(2637-6469) |
| Samoa | | 286(172-431) | | 517(301-791) | 780(499-1138) | | 1033(617-1553) | | | | 1079(683-1581) | 3695(2271-5494) |
| St. Vincent and the Grenadines | | 234(148-357) | | 403(239-628) | 627(391-911) | | 863(511-1286) | | | | 972(614-1446) | 3100(1902-4629) |
| Kiribati | | 187(116-286) | | 263(153-409) | 406(254-593) | | 559(329-839) | | | | 576(363-852) | 1991(1215-2979) |
| Tonga | | 143(89-220) | | 246(145-378) | 380(239-548) | | 465(290-689) | | | | 508(324-741) | 1742(1086-2576) |
| TOTAL | | 23042826  (14827446  -34147101) | | 38677401  (23821729  -58860922) | 52986831  (34243870  -76176942) | | 60425000  (37177577  -87951963) | | | | 62251159  (40309593  -89626816) | 237383217  (150380215  -346763743) |
| Captions: Locations vary according to the GBD 2019 study of Low-and Middle-Income Countries. Ranked vary according to the number of cases of Presbyopia people aged 40-64 years old from more to less. The number of presbyopia is determined by number of cases and mortality, which are according to the GBD 2019 study. The number of cases of presbyopia are given inform with 95% confidence interval. | | | | | | | | | | | | |

**Table S2. ER、LPR、Per Capital GDP、GDP and population(40-64 years old) in LMICs in 2019.**

| **Location** | **ER** | **LPR** | **Per Capital GDP** | **GDP** | **Population of 40-64y** |
| --- | --- | --- | --- | --- | --- |
| Afghanistan | 0.42 | 0.49 | 494.18 | 18799450742.78 | 5165283 |
| Albania | 0.53 | 0.6 | 5395.66 | 15400242874.88 | 876939 |
| Algeria | 0.35 | 0.43 | 3989.67 | 171767403748.19 | 10332692 |
| Angola | 0.7 | 0.77 | 2809.63 | 89417190341.25 | 4245985 |
| Argentina | 0.49 | 0.62 | 10056.64 | 451932356085.84 | 12070297 |
| Armenia | 0.4 | 0.53 | 4604.65 | 13619291361.28 | 930802 |
| Azerbaijan | 0.59 | 0.67 | 4805.75 | 48174235294.12 | 3092315 |
| Bangladesh | 0.53 | 0.59 | 1855.74 | 302571320445.75 | 36559914 |
| Belarus | 0.6 | 0.59 | 6837.72 | 64409647193.80 | 3362482 |
| Belize | 0.57 | 0.65 | 5078.81 | 1982518540.61 | 87978 |
| Benin | 0.68 | 0.71 | 1219.52 | 14391686632.78 | 1669041 |
| Bhutan | 0.63 | 0.67 | 3322.86 | 2535657068.97 | 162709 |
| Bolivia | 0.63 | 0.72 | 3552.07 | 40895322843.79 | 2521610 |
| Bosnia and Herzegovina | 0.38 | 0.47 | 6119.76 | 20201323260.07 | 1218586 |
| Botswana | 0.57 | 0.71 | 7203.06 | 16593720655.64 | 485043 |
| Brazil | 0.51 | 0.65 | 8897.55 | 1877824273720.78 | 62515651 |
| Bulgaria | 0.52 | 0.57 | 9879.27 | 68915416141.96 | 2479006 |
| Burkina Faso | 0.62 | 0.67 | 796.12 | 16178162030.07 | 3118327 |
| Burundi | 0.78 | 0.79 | 228.21 | 2631434363.23 | 1614204 |
| Cabo Verde | 0.49 | 0.6 | 3603.78 | 1981845740.71 | 123594 |
| Cambodia | 0.79 | 0.82 | 1643.12 | 27089389786.98 | 3571427 |
| Cameroon | 0.72 | 0.76 | 1533.10 | 39670977332.73 | 4161339 |
| Central African Republic | 0.67 | 0.72 | 467.91 | 2220307368.70 | 832117 |
| Chad | 0.67 | 0.71 | 709.54 | 11314951342.78 | 1896551 |
| China | 0.63 | 0.68 | 10143.84 | 14279937467431.00 | 521510751 |
| Colombia | 0.53 | 0.68 | 6424.98 | 323429888934.26 | 13009033 |
| Comoros | 0.41 | 0.46 | 1401.54 | 1192559739.37 | 146795 |
| Congo, Dem. Rep | 0.59 | 0.64 | 580.72 | 50400747050.50 | 12670487 |
| Congo, Rep. | 0.6 | 0.64 | 2369.73 | 12750339022.95 | 1002150 |
| Costa Rica | 0.6 | 0.64 | 12693.83 | 64072870931.60 | 1305039 |
| Côte d'Ivoire | 0.6 | 0.54 | 2276.33 | 58539424929.72 | 4036477 |
| Cuba | 0.48 | 0.54 | 9125.88 | 103428000000.00 | 4195421 |
| Djibouti | 0.52 | 0.6 | 3414.92 | 3324615549.09 | 239880 |
| Dominican Republic | 0.56 | 0.67 | 8282.12 | 88941299733.50 | 2568613 |
| Ecuador | 0.59 | 0.68 | 6222.52 | 108108009000.00 | 4053201 |
| Egypt, Arab Rep. | 0.39 | 0.45 | 3019.09 | 303080865603.65 | 20839514 |
| El Salvador | 0.49 | 0.59 | 4167.73 | 26896660000.00 | 1438267 |
| Equatorial Guinea | 0.55 | 0.62 | 8419.93 | 11417278044.99 | 171283 |
| Eritrea | 0.69 | 0.79 | 1600.00 | 9835836800.00 | 1035181 |
| Eswatini | 0.39 | 0.52 | 3915.64 | 4495679480.97 | 184725 |
| Ethiopia | 0.74 | 0.79 | 855.76 | 95912590628.14 | 14092323 |
| Fiji | 0.53 | 0.58 | 6175.89 | 5496264766.37 | 233878 |
| Gabon | 0.41 | 0.53 | 7767.00 | 16874405839.79 | 349175 |
| Gambia | 0.52 | 0.59 | 772.05 | 1812529104.62 | 306475 |
| Georgia | 0.56 | 0.64 | 4697.98 | 17477255562.26 | 1187768 |
| Ghana | 0.63 | 0.68 | 2246.63 | 68337537815.77 | 5461201 |
| Guatemala | 0.54 | 0.63 | 4638.63 | 77020015201.32 | 3104580 |
| Guinea | 0.57 | 0.61 | 1058.14 | 13513809258.21 | 1684850 |
| Guinea-Bissau | 0.67 | 0.72 | 749.45 | 1439638443.38 | 254804 |
| Guyana | 0.43 | 0.55 | 6609.51 | 5173760191.85 | 191348 |
| Haiti | 0.55 | 0.68 | 1312.77 | 14785839382.90 | 2305460 |
| Honduras | 0.54 | 0.69 | 2556.46 | 24915522849.15 | 1802066 |
| India | 0.43 | 0.49 | 2100.75 | 2870504096717.77 | 325223030 |
| Indonesia | 0.64 | 0.68 | 4135.20 | 1119091259074.62 | 73044169 |
| Iran, Islamic Rep | 0.38 | 0.45 | 3114.62 | 258245497664.39 | 22740338 |
| Iraq | 0.36 | 0.43 | 5980.63 | 235097182233.50 | 8045878 |
| Jamaica | 0.59 | 0.67 | 5369.50 | 15830768549.89 | 738243 |
| Jordan | 0.31 | 0.39 | 4405.49 | 44502895915.49 | 2455109 |
| Kazakhstan | 0.62 | 0.69 | 9812.60 | 181667190075.54 | 5049734 |
| Kenya | 0.7 | 0.74 | 1912.65 | 100555485831.94 | 7890507 |
| Kiribati | 0.33 | 0.36 | 1601.86 | 188391770.64 | 23822 |
| Korea, Dem. People's Rep | 0.77 | 0.81 | 1700.00 | 40000000000.00 | 8643150 |
| Kyrgyz Republic | 0.51 | 0.59 | 1374.03 | 8871026074.20 | 1470760 |
| Lao PDR | 0.77 | 0.78 | 2635.80 | 18897252232.58 | 1454001 |
| Lebanon | 0.42 | 0.48 | 7578.17 | 51953744507.41 | 1268093 |
| Lesotho | 0.5 | 0.68 | 1113.37 | 2366213068.58 | 384845 |
| Liberia | 0.7 | 0.76 | 672.34 | 3319596500.00 | 798317 |
| Libya | 0.39 | 0.5 | 7685.95 | 52091152228.34 | 1883878 |
| Madagascar | 0.82 | 0.86 | 522.99 | 14104664514.87 | 4174606 |
| Malawi | 0.71 | 0.76 | 591.85 | 11025357837.61 | 2398906 |
| Malaysia | 0.61 | 0.65 | 11432.82 | 365276282438.14 | 7680711 |
| Maldives | 0.63 | 0.7 | 10561.61 | 5607762625.15 | 116585 |
| Mali | 0.64 | 0.69 | 879.04 | 17280251193.95 | 2791531 |
| Mauritania | 0.4 | 0.45 | 1743.30 | 7889662452.17 | 632438 |
| Mauritius | 0.52 | 0.58 | 11097.17 | 14045808843.22 | 441967 |
| Mexico | 0.54 | 0.61 | 9950.45 | 1269433932805.91 | 32781378 |
| Moldova | 0.38 | 0.42 | 4491.69 | 11970233939.88 | 1270458 |
| Mongolia | 0.6 | 0.63 | 4404.85 | 14206359006.81 | 884399 |
| Montenegro | 0.45 | 0.56 | 8910.65 | 5542674317.06 | 207864 |
| Morocco | 0.38 | 0.45 | 3235.00 | 119870439113.66 | 9563343 |
| Mozambique | 0.74 | 0.78 | 506.64 | 15384701797.99 | 3770821 |
| Myanmar | 0.56 | 0.6 | 1271.11 | 68697761477.18 | 14071280 |
| Namibia | 0.47 | 0.59 | 5009.69 | 12496781719.78 | 435427 |
| Nepal | 0.74 | 0.82 | 1194.96 | 34186180694.93 | 6298296 |
| Nicaragua | 0.61 | 0.67 | 1926.70 | 12611218627.06 | 1360373 |
| Niger | 0.71 | 0.72 | 554.10 | 12916455161.11 | 2612446 |
| Nigeria | 0.49 | 0.55 | 2229.86 | 448120428858.77 | 31362635 |
| North Macedonia | 0.42 | 0.55 | 6070.39 | 12606338448.55 | 741258 |
| Pakistan | 0.48 | 0.53 | 1288.56 | 279056608888.64 | 37785724 |
| Panama | 0.56 | 0.67 | 15774.25 | 66984427150.32 | 1055222 |
| Papua New Guinea | 0.45 | 0.47 | 2820.31 | 24751344552.21 | 1805921 |
| Paraguay | 0.65 | 0.73 | 5380.96 | 37906944074.61 | 1534224 |
| Peru | 0.61 | 0.78 | 7027.61 | 228470919605.67 | 8480064 |
| Philippines | 0.55 | 0.6 | 3485.34 | 376823278560.85 | 24730297 |
| Romania | 0.52 | 0.55 | 12899.35 | 249881592298.07 | 6893079 |
| Russian Federation | 0.58 | 0.61 | 11497.65 | 1687448525466.61 | 49896707 |
| Rwanda | 0.79 | 0.84 | 820.15 | 10355974217.37 | 2008311 |
| Samoa | 0.39 | 0.44 | 4322.87 | 852007112.72 | 43747 |
| São Tomé and Principe | 0.48 | 0.58 | 1987.58 | 427425039.68 | 36847 |
| Senegal | 0.41 | 0.46 | 1430.15 | 23306213742.36 | 2338881 |
| Serbia | 0.49 | 0.55 | 7417.20 | 51514222381.84 | 2839067 |
| Sierra Leone | 0.54 | 0.58 | 521.75 | 4076578542.56 | 1182843 |
| Solomon Islands | 0.83 | 0.84 | 2344.05 | 1570093229.26 | 117069 |
| Somalia | 0.41 | 0.48 | 419.39 | 6476674591.87 | 2561020 |
| South Africa | 0.37 | 0.56 | 6624.76 | 387934574098.17 | 13096790 |
| South Sudan | 0.61 | 0.72 | 1600.00 | 17574518400.00 | 1442621 |
| Sri Lanka | 0.49 | 0.53 | 3852.27 | 83990946939.47 | 6475139 |
| St. Lucia | 0.58 | 0.72 | 11591.08 | 2118791555.56 | 57132 |
| St. Vincent and the Grenadines | 0.51 | 0.67 | 7460.15 | 825040740.74 | 34642 |
| Sudan | 0.39 | 0.49 | 610.93 | 26155799324.87 | 6412082 |
| Suriname | 0.44 | 0.52 | 6853.69 | 3984483762.37 | 166151 |
| Syrian Arab Republic | 0.39 | 0.44 | 1334.37 | 22777882165.11 | 4007097 |
| Tajikistan | 0.36 | 0.4 | 890.54 | 8300784856.88 | 1903250 |
| Tanzania | 0.81 | 0.83 | 1085.88 | 61136873692.40 | 8292703 |
| Thailand | 0.65 | 0.67 | 7817.01 | 544263840039.17 | 26437150 |
| Timor-Leste | 0.64 | 0.67 | 1583.71 | 2047931700.00 | 222025 |
| Togo | 0.55 | 0.59 | 893.35 | 7220395247.74 | 1340158 |
| Tonga | 0.44 | 0.47 | 4903.01 | 512350059.42 | 21282 |
| Tunisia | 0.38 | 0.46 | 3574.65 | 41804581484.37 | 3446473 |
| Turkey | 0.43 | 0.53 | 9121.52 | 761004425605.41 | 24114718 |
| Turkmenistan | 0.54 | 0.58 | 7612.04 | 45231428571.43 | 1268742 |
| Uganda | 0.65 | 0.7 | 798.59 | 35353060634.20 | 4910617 |
| Ukraine | 0.48 | 0.54 | 3661.46 | 153882982016.28 | 15464359 |
| Uzbekistan | 0.56 | 0.62 | 1784.01 | 59907674027.47 | 7935361 |
| Vanuatu | 0.67 | 0.7 | 3116.30 | 934521604.60 | 52573 |
| Vietnam | 0.74 | 0.77 | 2715.28 | 261921244843.17 | 28837640 |
| Yemen, Rep. | 0.33 | 0.38 | 631.49 | 22581081993.95 | 4594406 |
| Zambia | 0.65 | 0.75 | 1305.00 | 23308667781.23 | 2469267 |
| Zimbabwe | 0.76 | 0.83 | 1316.74 | 19284289739.05 | 2330852 |
| Captions: Location according to the GBD 2019 study of Low-and Middle-Income Countries. ER= employment rate，according to the International Labor Organization. LPR= Labor force participation rate，according to the International Labor Organization. GDP=gross domestic product. GDP per capital=gross domestic product per capital，derived from World Bank open data，GDP was measured by current US $，and several data were derived from CIA’s World Factbook when GDP data was not available. | | | | | |

**Table S3. Mortality in 2019 by age group.**

| **Location** | **40-44Y** | **45-49Y** | **50-54Y** | **55-59Y** | **60-64Y** |
| --- | --- | --- | --- | --- | --- |
| Afghanistan | 0.03 | 0.05 | 0.07 | 0.10 | 0.14 |
| Albania | 0.01 | 0.01 | 0.01 | 0.02 | 0.04 |
| Algeria | 0.01 | 0.01 | 0.02 | 0.03 | 0.05 |
| Angola | 0.03 | 0.04 | 0.06 | 0.08 | 0.12 |
| Argentina | 0.01 | 0.02 | 0.03 | 0.04 | 0.06 |
| Armenia | 0.01 | 0.02 | 0.03 | 0.04 | 0.07 |
| Azerbaijan | 0.01 | 0.02 | 0.03 | 0.05 | 0.09 |
| Bangladesh | 0.01 | 0.02 | 0.03 | 0.04 | 0.07 |
| Belarus | 0.02 | 0.03 | 0.04 | 0.06 | 0.08 |
| Belize | 0.02 | 0.02 | 0.03 | 0.05 | 0.07 |
| Benin | 0.02 | 0.03 | 0.05 | 0.07 | 0.11 |
| Bhutan | 0.01 | 0.02 | 0.03 | 0.04 | 0.07 |
| Bolivia | 0.01 | 0.02 | 0.03 | 0.05 | 0.08 |
| Bosnia and Herzegovina | 0.01 | 0.01 | 0.02 | 0.04 | 0.06 |
| Botswana | 0.06 | 0.07 | 0.09 | 0.11 | 0.14 |
| Brazil | 0.01 | 0.02 | 0.03 | 0.04 | 0.06 |
| Bulgaria | 0.01 | 0.02 | 0.04 | 0.06 | 0.09 |
| Burkina Faso | 0.03 | 0.04 | 0.06 | 0.08 | 0.12 |
| Burundi | 0.03 | 0.04 | 0.06 | 0.09 | 0.13 |
| Cabo Verde | 0.01 | 0.02 | 0.03 | 0.05 | 0.07 |
| Cambodia | 0.02 | 0.03 | 0.04 | 0.06 | 0.10 |
| Cameroon | 0.04 | 0.05 | 0.06 | 0.08 | 0.12 |
| Central African Republic | 0.07 | 0.09 | 0.12 | 0.16 | 0.20 |
| Chad | 0.03 | 0.04 | 0.06 | 0.08 | 0.12 |
| China | 0.01 | 0.01 | 0.02 | 0.03 | 0.05 |
| Colombia | 0.01 | 0.01 | 0.02 | 0.02 | 0.04 |
| Comoros | 0.02 | 0.03 | 0.04 | 0.06 | 0.09 |
| Congo, Dem. Rep | 0.03 | 0.04 | 0.06 | 0.08 | 0.12 |
| Congo, Rep. | 0.03 | 0.05 | 0.06 | 0.09 | 0.12 |
| Costa Rica | 0.01 | 0.01 | 0.02 | 0.03 | 0.04 |
| Côte d'Ivoire | 0.03 | 0.04 | 0.06 | 0.08 | 0.11 |
| Cuba | 0.01 | 0.01 | 0.02 | 0.04 | 0.06 |
| Djibouti | 0.03 | 0.04 | 0.05 | 0.07 | 0.10 |
| Dominican Republic | 0.02 | 0.02 | 0.04 | 0.05 | 0.08 |
| Ecuador | 0.01 | 0.02 | 0.02 | 0.04 | 0.05 |
| Egypt, Arab Rep. | 0.01 | 0.02 | 0.04 | 0.07 | 0.11 |
| El Salvador | 0.02 | 0.02 | 0.03 | 0.04 | 0.06 |
| Equatorial Guinea | 0.04 | 0.05 | 0.06 | 0.07 | 0.10 |
| Eritrea | 0.04 | 0.05 | 0.07 | 0.10 | 0.14 |
| Eswatini | 0.08 | 0.09 | 0.11 | 0.13 | 0.15 |
| Ethiopia | 0.02 | 0.03 | 0.04 | 0.06 | 0.09 |
| Fiji | 0.02 | 0.03 | 0.06 | 0.08 | 0.13 |
| Gabon | 0.03 | 0.04 | 0.06 | 0.08 | 0.11 |
| Gambia | 0.03 | 0.04 | 0.06 | 0.08 | 0.11 |
| Georgia | 0.02 | 0.03 | 0.04 | 0.06 | 0.08 |
| Ghana | 0.03 | 0.04 | 0.05 | 0.07 | 0.11 |
| Guatemala | 0.02 | 0.03 | 0.04 | 0.05 | 0.07 |
| Guinea | 0.03 | 0.04 | 0.06 | 0.09 | 0.13 |
| Guinea-Bissau | 0.04 | 0.06 | 0.08 | 0.11 | 0.15 |
| Guyana | 0.03 | 0.04 | 0.06 | 0.08 | 0.12 |
| Haiti | 0.03 | 0.04 | 0.06 | 0.08 | 0.12 |
| Honduras | 0.02 | 0.02 | 0.04 | 0.06 | 0.09 |
| India | 0.02 | 0.02 | 0.04 | 0.06 | 0.08 |
| Indonesia | 0.01 | 0.02 | 0.04 | 0.06 | 0.09 |
| Iran, Islamic Rep | 0.01 | 0.01 | 0.02 | 0.03 | 0.05 |
| Iraq | 0.01 | 0.02 | 0.03 | 0.05 | 0.08 |
| Jamaica | 0.01 | 0.02 | 0.03 | 0.04 | 0.06 |
| Jordan | 0.01 | 0.01 | 0.02 | 0.03 | 0.05 |
| Kazakhstan | 0.02 | 0.03 | 0.04 | 0.06 | 0.09 |
| Kenya | 0.03 | 0.05 | 0.06 | 0.08 | 0.11 |
| Kiribati | 0.05 | 0.07 | 0.10 | 0.14 | 0.18 |
| Korea, Dem. People's Rep | 0.02 | 0.02 | 0.04 | 0.06 | 0.09 |
| Kyrgyz Republic | 0.02 | 0.02 | 0.03 | 0.05 | 0.08 |
| Lao PDR | 0.02 | 0.03 | 0.04 | 0.06 | 0.10 |
| Lebanon | 0.01 | 0.01 | 0.02 | 0.04 | 0.06 |
| Lesotho | 0.11 | 0.14 | 0.15 | 0.17 | 0.20 |
| Liberia | 0.03 | 0.04 | 0.05 | 0.07 | 0.10 |
| Libya | 0.01 | 0.02 | 0.03 | 0.04 | 0.07 |
| Madagascar | 0.03 | 0.04 | 0.05 | 0.08 | 0.12 |
| Malawi | 0.03 | 0.05 | 0.06 | 0.08 | 0.11 |
| Malaysia | 0.01 | 0.02 | 0.03 | 0.05 | 0.07 |
| Maldives | 0.01 | 0.01 | 0.01 | 0.02 | 0.04 |
| Mali | 0.03 | 0.03 | 0.05 | 0.07 | 0.10 |
| Mauritania | 0.02 | 0.02 | 0.03 | 0.05 | 0.08 |
| Mauritius | 0.01 | 0.02 | 0.03 | 0.05 | 0.07 |
| Mexico | 0.02 | 0.02 | 0.03 | 0.04 | 0.06 |
| Moldova | 0.02 | 0.03 | 0.04 | 0.06 | 0.08 |
| Mongolia | 0.03 | 0.04 | 0.06 | 0.09 | 0.13 |
| Montenegro | 0.01 | 0.02 | 0.03 | 0.05 | 0.07 |
| Morocco | 0.01 | 0.02 | 0.03 | 0.05 | 0.08 |
| Mozambique | 0.06 | 0.07 | 0.09 | 0.11 | 0.15 |
| Myanmar | 0.02 | 0.03 | 0.04 | 0.06 | 0.09 |
| Namibia | 0.04 | 0.06 | 0.07 | 0.09 | 0.11 |
| Nepal | 0.01 | 0.02 | 0.04 | 0.06 | 0.09 |
| Nicaragua | 0.01 | 0.02 | 0.03 | 0.04 | 0.07 |
| Niger | 0.02 | 0.03 | 0.05 | 0.07 | 0.11 |
| Nigeria | 0.02 | 0.03 | 0.04 | 0.06 | 0.09 |
| North Macedonia | 0.01 | 0.02 | 0.03 | 0.05 | 0.07 |
| Pakistan | 0.02 | 0.03 | 0.05 | 0.07 | 0.11 |
| Panama | 0.01 | 0.01 | 0.02 | 0.03 | 0.04 |
| Papua New Guinea | 0.03 | 0.04 | 0.06 | 0.09 | 0.13 |
| Paraguay | 0.01 | 0.02 | 0.03 | 0.04 | 0.06 |
| Peru | 0.01 | 0.01 | 0.02 | 0.03 | 0.04 |
| Philippines | 0.02 | 0.03 | 0.04 | 0.06 | 0.08 |
| Romania | 0.01 | 0.02 | 0.04 | 0.05 | 0.07 |
| Russian Federation | 0.02 | 0.03 | 0.04 | 0.06 | 0.08 |
| Rwanda | 0.02 | 0.03 | 0.04 | 0.06 | 0.09 |
| Samoa | 0.02 | 0.03 | 0.05 | 0.07 | 0.10 |
| São Tomé and Principe | 0.02 | 0.02 | 0.04 | 0.06 | 0.09 |
| Senegal | 0.02 | 0.03 | 0.04 | 0.06 | 0.10 |
| Serbia | 0.01 | 0.02 | 0.03 | 0.05 | 0.07 |
| Sierra Leone | 0.03 | 0.04 | 0.06 | 0.08 | 0.12 |
| Solomon Islands | 0.06 | 0.09 | 0.12 | 0.16 | 0.21 |
| Somalia | 0.04 | 0.06 | 0.08 | 0.12 | 0.16 |
| South Africa | 0.05 | 0.05 | 0.06 | 0.07 | 0.10 |
| South Sudan | 0.03 | 0.04 | 0.05 | 0.07 | 0.10 |
| Sri Lanka | 0.01 | 0.02 | 0.02 | 0.03 | 0.05 |
| St. Lucia | 0.02 | 0.02 | 0.03 | 0.04 | 0.06 |
| St. Vincent and the Grenadines | 0.02 | 0.03 | 0.04 | 0.05 | 0.07 |
| Sudan | 0.02 | 0.02 | 0.03 | 0.05 | 0.08 |
| Suriname | 0.02 | 0.02 | 0.04 | 0.05 | 0.08 |
| Syrian Arab Republic | 0.01 | 0.02 | 0.03 | 0.05 | 0.08 |
| Tajikistan | 0.01 | 0.02 | 0.03 | 0.06 | 0.10 |
| Tanzania | 0.02 | 0.03 | 0.05 | 0.06 | 0.10 |
| Thailand | 0.02 | 0.02 | 0.02 | 0.03 | 0.05 |
| Timor-Leste | 0.02 | 0.02 | 0.04 | 0.06 | 0.09 |
| Togo | 0.03 | 0.04 | 0.06 | 0.08 | 0.11 |
| Tonga | 0.02 | 0.03 | 0.04 | 0.06 | 0.09 |
| Tunisia | 0.01 | 0.01 | 0.02 | 0.03 | 0.05 |
| Turkey | 0.01 | 0.01 | 0.02 | 0.03 | 0.05 |
| Turkmenistan | 0.02 | 0.03 | 0.04 | 0.06 | 0.10 |
| Uganda | 0.03 | 0.04 | 0.05 | 0.07 | 0.10 |
| Ukraine | 0.02 | 0.03 | 0.04 | 0.05 | 0.08 |
| Uzbekistan | 0.02 | 0.03 | 0.04 | 0.07 | 0.12 |
| Vanuatu | 0.04 | 0.05 | 0.07 | 0.10 | 0.14 |
| Vietnam | 0.01 | 0.02 | 0.03 | 0.05 | 0.07 |
| Yemen, Rep. | 0.02 | 0.03 | 0.04 | 0.06 | 0.10 |
| Zambia | 0.04 | 0.06 | 0.07 | 0.10 | 0.13 |
| Zimbabwe | 0.05 | 0.07 | 0.09 | 0.11 | 0.15 |
| Captions: Location according to the GBD 2019 study of Low-and Middle-Income Countries. Mortality according to the GBD 2019 study. | | | | | |

**Table S4. Number of presbyopia cases followed up to retirement by age group.**

| **Location** | **40-44Y** | | | | | | | | | | | | | | | | | **45-49Y** | **50-54Y** | | **55-59Y** | | **60-64Y** |
| --- | --- | --- | --- | --- | --- | --- | --- | --- | --- | --- | --- | --- | --- | --- | --- | --- | --- | --- | --- | --- | --- | --- | --- |
| Afghanistan | 16897(9874-26076) | | | | | | | | | | | | | | | | | 38926(22118-60149) | 49218(30180-72233) | | 39522(23388-59945) | | 35887(22913-53092) |
| Albania | 4329(2646-6731) | | | | | | | | | | | | | | | | | 8945(5401-13972) | 17214(10863-24967) | | 29236(16768-43735) | | 38915(23969-56893) |
| Algeria | 38131(22710-58841) | | | | | | | | | | | | | | | | | 59683(34010-91942) | 90352(55876-131635) | | 119175(69180-180615) | | 144127(91421-213245) |
| Angola | 46324(28970-69647) | | | | | | | | | | | | | | | | | 66765(40234-101900) | 93238(59428-136383) | | 118098(70414-179853) | | 120511(77675-179096) |
| Argentina | 7839(4208-12948) | | | | | | | | | | | | | | | | | 11285(5851-18488) | 16176(8922-25018) | | 23659(12187-37675) | | 33040(18881-52393) |
| Armenia | 4996(3062-7610) | | | | | | | | | | | | | | | | | 8132(4727-12645) | 14677(9162-21306) | | 29267(17442-44995) | | 41417(25738-61039) |
| Azerbaijan | 16287(10001-24727) | | | | | | | | | | | | | | | | | 29293(17262-45688) | 55133(34474-79909) | | 90191(54404-133498) | | 99934(64610-144788) |
| Bangladesh | 808310  (514935-1200284) | | | | | | | | | | | | | | | | | 1234227  (733088-1913552) | 1537325  (963422-2266826) | | 1748927  (1054515-2566491) | | 1773950  (1115791-2630022) |
| Belarus | 23933(14946-35722) | | | | | | | | | | | | | | | | | 44225(26408-66982) | 77430(48785-112493) | | 144669(86554-219687) | | 198077(128169-290475) |
| Belize | 616(379-931) | | | | | | | | | | | | | | | | | 989(590-1519) | 1360(869-1969) | | 1747(1048-2570) | | 1862(1160-2713) |
| Benin | 28155(17850-41716) | | | | | | | | | | | | | | | | | 40337(24971-61099) | 47793(30386-69620) | | 52893(32564-77675) | | 52778(33906-76657) |
| Bhutan | 3901(2483-5831) | | | | | | | | | | | | | | | | | 5614(3403-8594) | 6812(4277-9956) | | 7673(4532-11315) | | 7743(4790-11376) |
| Bolivia | 13463(8293-20660) | | | | | | | | | | | | | | | | | 20728(12160-32332) | 30726(19437-44965) | | 42983(25116-65087) | | 54581(34446-79897) |
| Bosnia and Herzegovina | | | | | | | | | | | | | | 6433(3845-9841) | | | | 11338(6697-17560) | 21079(13254-30581) | | 36255(20910-53986) | | 51722(32271-76291) |
| Botswana | 13196(8474-19614) | | | | | | | | | | | | | | | | | 18175(11309-28155) | 21025(13426-30667) | | 25367(15447-36997) | | 26256(16780-37898) |
| Brazil | 188831  (113924-290581) | | | | | | | | | | | | | | | | | 304230  (177283-476133) | 517140  (316055-748747) | | 769419  (449418-1157500) | | 1010634  (629908-1456780) |
| Bulgaria | 12315(7429-18940) | | | | | | | | | | | | | | | | | 22689(13377-34873) | 37628(23586-55136) | | 61991(36919-91564) | | 96210(61224-138804) |
| Burkina Faso | | | | 52824(33608-78369) | | | | | | | | | | | | | | 75827(46424-114767) | 93929(60229-135065) | | 108414(68156-158757) | | 108937(70602-156111) |
| Burundi | 23131(14648-34983) | | | | | | | | | | | | | | | | | 32899(19796-49533) | 45373(28779-65185) | | 58623(36292-85522) | | 64336(41360-94508) |
| Cabo Verde | 1710(1071-2533) | | | | | | | | | | | | | | | | | 2644(1568-4133) | 3783(2380-5513) | | 4692(2898-6796) | | 4867(3115-7103) |
| Cambodia | 20722(12598-31779) | | | | | | | | | | | | | | | | | 33704(20236-51810) | 58447(37611-85978) | | 82508(50482-125726) | | 96593(62214-142383) |
| Cameroon | 61060(39226-92924) | | | | | | | | | | | | | | | | | 87103(52094-132794) | 107508(68585-155657) | | 125499(76268-184022) | | 126465(81461-183443) |
| Central African Republic | | | | 6652(4130-9953) | | | | | | | | | | | | | | 10827(6626-16533) | 15806(9696-22827) | | 20396(11891-30676) | | 23747(14666-35004) |
| Chad | 31281(19665-47529) | | | | | | | | | | | | | | | | | 46340(28071-72684) | 56095(35363-83310) | | 63260(39467-91240) | | 63223(40605-91086) |
| China | 4473588  (2897925-6621984) | | | | | | | | | | | | | | | | | 10225537  (6358453-15583928) | 17204000  (11215522-24569824) | | 19701384  (12216343-28679966) | | 21595280  (14055744-31027659) |
| Colombia | 73927(44221-111538) | | | | | | | | | | | | | | | | | 129259(76121-199349) | 224237(139531-324303) | | 323392(190008-485254) | | 385780(245613-560780) |
| Comoros | 1990(1266-3002) | | | | | | | | | | | | | | | | | 3229(1936-4971) | 4331(2754-6197) | | 5142(3108-7599) | | 5524(3566-8078) |
| Congo, Dem. Rep | | | | | | | | | | | | | 136713(84797-208117) | | | | | 209813(123998-323163) | 293912(185913-432261) | | 373082(226384-560798) | | 401446(259027-589597) |
| Congo, Rep. | | | | 9997(6073-15312) | | | | | | | | | | | | | | 15287(8877-23766) | 19697(12472-28307) | | 23414(14278-34988) | | 24629(15814-35885) |
| Costa Rica | 7369(4427-11229) | | | | | | | | | | | | | | | | | 12145(7194-18521) | 21316(13509-30781) | | 31697(19237-47600) | | 38156(24873-56173) |
| Côte d'Ivoire | | | | | | 65265(41300-99271) | | | | | | | | | | | | 90711(53874-140980) | 107853(66902-157327) | | 121846(73987-180762) | | 117970(75441-175115) |
| Cuba | 18511(11242-27983) | | | | | | | | | | | | | | | | | 45988(26451-70646) | 79173(50677-114652) | | 105755(65361-157471) | | 100977(65809-147344) |
| Djibouti | 3490(2176-5242) | | | | | | | | | | | | | | | | | 5106(3029-7854) | 6362(4075-9281) | | 7230(4341-10742) | | 7380(4617-10662) |
| Dominican Republic | | | | | | | | | | | | | | | | 17340(10752-26367) | | 28031(16575-43100) | 40638(25640-59575) | | 52308(31489-79417) | | 59016(37458-86559) |
| Ecuador | 20721(12343-31606) | | | | | | | | | | | | | | | | | 33384(19748-52072) | 50676(30720-73944) | | 72249(42158-108901) | | 85613(52603-123002) |
| Egypt, Arab Rep. | | | | | | | | | 66646(39068-102515) | | | | | | | | | 100333(57296-155349) | 159946(97266-234769) | | 227236(128984-344728) | | 280305(171512-417091) |
| El Salvador | 8920(5554-13614) | | | | | | | | | | | | | | | | | 14679(8770-22671) | 22869(14314-33373) | | 31642(19441-47960) | | 39369(25111-57901) |
| Equatorial Guinea | | | | | | | | | | | | 1773(1127-2644) | | | | | | 2567(1527-4016) | 3372(2074-4970) | | 3992(2313-6064) | | 4321(2708-6355) |
| Eritrea | 15645(10260-23324) | | | | | | | | | | | | | | | | | 23963(14835-36422) | 29179(18846-42023) | | 32511(20142-47029) | | 32240(20162-46565) |
| Eswatini | 4614(2969-6828) | | | | | | | | | | | | | | | | | 6546(3951-9997) | 8066(5111-11680) | | 9491(5807-14109) | | 10512(6779-15445) |
| Ethiopia | 128241(79716-194210) | | | | | | | | | | | | | | | | | 177609(106164-270306) | 234345(150576-336602) | | 291788(178939-429755) | | 343867(220023-494859) |
| Fiji | 1083(655-1675) | | | | | | | | | | | | | | | | | 1780(1049-2747) | 3107(1902-4578) | | 4731(2744-7223) | | 5569(3494-8204) |
| Gabon | 3124(1936-4799) | | | | | | | | | | | | | | | | | 5016(2999-7673) | 7169(4508-10290) | | 9346(5627-13820) | | 10180(6486-14727) |
| Gambia | 5009(3171-7503) | | | | | | | | | | | | | | | | | 6869(4123-10726) | 8245(5256-12107) | | 9217(5676-13492) | | 9810(6229-14173) |
| Georgia | 5760(3438-9112) | | | | | | | | | | | | | | | | | 10555(6258-16658) | 18792(11585-27672) | | 34719(20034-52276) | | 49268(30676-71427) |
| Ghana | 68502(42368-105093) | | | | | | | | | | | | | | | | | 101253(61119-155566) | 127341(82114-182794) | | 149942(94266-216805) | | 162604(106122-229182) |
| Guatemala | 21964(13639-35007) | | | | | | | | | | | | | | | | | 31750(18850-50727) | 45381(28585-65853) | | 63167(37673-93767) | | 78879(50411-114891) |
| Guinea | 25661(16324-38598) | | | | | | | | | | | | | | | | | 38209(23201-57899) | 47436(30314-68867) | | 56510(34332-83010) | | 61714(39478-90947) |
| Guinea-Bissau | | | | | | 3757(2390-5637) | | | | | | | | | | | | 5285(3158-8025) | 6526(4197-9447) | | 7572(4726-11286) | | 7892(5088-11549) |
| Guyana | 1042(639-1615) | | | | | | | | | | | | | | | | | 1761(1013-2806) | 2696(1670-4001) | | 3759(2199-5662) | | 4362(2734-6473) |
| Haiti | 16732(10083-25156) | | | | | | | | | | | | | | | | | 24097(14121-36581) | 32828(20845-47253) | | 42794(25973-63140) | | 50171(31518-73567) |
| Honduras | 12173(7518-18658) | | | | | | | | | | | | | | | | | 18708(10834-28857) | 26651(16492-38749) | | 36548(21599-54897) | | 44981(29063-67107) |
| India | 7553850  (4905991-11114305) | | | | | | | | | | | | | | | | | 11539247  (7147579-17497309) | 14175031  (9133172-20573958) | | 16124526  (9858204-23346813) | | 17190481  (11105196-24846733) |
| Indonesia | 255501  (154968-393130) | | | | | | | | | | | | | | | | | 424216  (247502-657113) | 668335  (415618-975429) | | 929726  (556760-1393828) | | 1144447  (719500-1670555) |
| Iran, Islamic Rep | 56053  (32892-87995) | | | | | | | | | | | | | | | | | 83717  (47620-130592) | 129403  (77266-190022) | | 171991  (97935-260913) | | 229782  (140839-333809) |
| Iraq | 28494(17096-44068) | | | | | | | | | | | | | | | | | 47358(27226-73860) | 62335(38682-92579) | | 77718(46707-117841) | | 92707(59188-135415) |
| Jamaica | 4303(2645-6650) | | | | | | | | | | | | | | | | | 7697(4548-11861) | 11777(7471-17161) | | 15947(9730-23689) | | 18459(12059-27281) |
| Jordan | 9094(5327-14141) | | | | | | | | | | | | | | | | | 14716(8385-23055) | 21361(12966-31522) | | 25398(14478-39485) | | 25918(15988-38367) |
| Kazakhstan | 28791(17517-43857) | | | | | | | | | | | | | | | | | 49815(29138-76631) | 80009(49342-116634) | | 129314(76868-191799) | | 155255(98138-225603) |
| Kenya | 61502(38651-93585) | | | | | | | | | | | | | | | | | 94193(56654-144721) | 133509(85275-191757) | | 176647(108337-262773) | | 204383(131769-296818) |
| Kiribati | 111(68-169) | | | | | | | | | | | | | | | | | 167(97-260) | 287(179-418) | | 457(269-686) | | 576(363-852) |
| Korea, Dem. People's Rep | | | | | | | | 71245(44440-107949) | | | | | | | | | | 159513(95659-248575) | 263320(166105-389698) | | 308491(187230-465804) | | 344701(214746-504290) |
| Kyrgyz Republic | | | | | | | | | | 9444(5792-13948) | | | | | | | | 16500(9809-25330) | 26600(16464-39103) | | 40234(23966-61210) | | 44975(28491-65902) |
| Lao PDR | 9115(5573-14105) | | | | | | | | | | | | | | | | | 14616(8574-22369) | 21904(13595-32119) | | 29587(17248-43988) | | 33719(21447-49460) |
| Lebanon | 4090(2461-6354) | | | | | | | | | | | | | | | | | 6927(3998-10782) | 10461(6561-15339) | | 15270(9381-23223) | | 17888(11406-26345) |
| Lesotho | 7253(4768-10648) | | | | | | | | | | | | | | | | | 10407(6426-15832) | 15073(9740-21877) | | 20349(12779-29572) | | 23241(15176-33325) |
| Liberia | 14063(8950-21060) | | | | | | | | | | | | | | | | | 21461(13022-33093) | 20561(13091-29695) | | 22472(13544-32976) | | 21189(13322-30637) |
| Libya | 7293(4300-11346) | | | | | | | | | | | | | | | | | 11163(6476-17567) | 14871(9277-21630) | | 16903(9814-25382) | | 18211(11321-26507) |
| Madagascar | | 57685(35357-85436) | | | | | | | | | | | | | | | | 87596(51916-133949) | 115105(73060-166652) | | 139451(85814-206277) | | 144221(93177-208662) |
| Malawi | 35708(22938-53392) | | | | | | | | | | | | | | | | | 51858(31114-77730) | 61479(39473-88446) | | 73813(45082-110194) | | 88235(56506-130089) |
| Malaysia | 41564(24731-63128) | | | | | | | | | | | | | | | | | 66127(38540-102598) | 105034(66297-153069) | | 151742(88272-227098) | | 189209(117088-276326) |
| Maldives | 950(578-1474) | | | | | | | | | | | | | | | | | 1307(748-2058) | 1743(1063-2537) | | 2178(1233-3261) | | 2268(1432-3255) |
| Mali | 46491(29608-68799) | | | | | | | | | | | | | | | | | 69287(42196-104457) | 87795(55928-127111) | | 102244(62239-150455) | | 104798(67458-152957) |
| Mauritania | 9851(6206-14596) | | | | | | | | | | | | | | | | | 14764(8928-22124) | 18477(11453-26431) | | 21694(12896-31567) | | 22665(14128-33487) |
| Mauritius | 2050(1259-3143) | | | | | | | | | | | | | | | | | 3409(1993-5324) | 6552(4048-9672) | | 10486(6297-15863) | | 13170(8349-19409) |
| Mexico | 148937(91484-228658) | | | | | | | | | | | | | | | | | 261104(154752-402766) | 415815(258407-601141) | 584547(342880-873927) | | 753750(473208-1093228) | |
| Moldova | 10343(6140-15499) | | | | | | | | | | | | | | | | | 17594(10382-26945) | 29304(18383-42540) | | 54364(32597-81485) | | 72093(45298-105742) |
| Mongolia | 5253(3224-8059) | | | | | | | | | | | | | | | | | 8762(5195-13579) | 13910(8785-19933) | | 19558(11623-29371) | | 20467(13132-29753) |
| Montenegro | | 1064(644-1629) | | | | | | | | | | | | | | | | 1886(1078-2943) | 3339(2090-4924) | | 5615(3465-8357) | | 8081(5149-11502) |
| Morocco | 32241(18909-52037) | | | | | | | | | | | | | | | | | 52294(29499-83270) | 83370(50783-124855) | | 125243(72363-190084) | | 156541(97719-229431) |
| Mozambique | | | 46398(29090-69123) | | | | | | | | | | | | | | | 73426(44696-113616) | 95615(60728-139238) | | 118254(72495-174687) | | 135631(87208-196756) |
| Myanmar | 79526(49219-120989) | | | | | | | | | | | | | | | | | 135797(79486-214304) | 215211(134462-311324) | | 302283(185257-449221) | | 363390(232435-521616) |
| Namibia | 12299(7959-18140) | | | | | | | | | | | | | | | | | 18232(11131-28013) | 21924(14244-32237) | | 24370(15248-35791) | | 24529(15800-35244) |
| Nepal | 325246(238725-413979) | | | | | | | | | | | | | | | | | 543959(383900-731714) | 629597(473243-803747) | | 624045(456870-777515) | | 563888(413227-699768) |
| Nicaragua | 9368(5834-14926) | | | | | | | | | | | | | | | | | 14518(8620-23135) | 21686(13654-31446) | | 29264(17447-43418) | | 34420(21995-50147) |
| Niger | 104113(77487-139544) | | | | | | | | | | | | | | | | | 146088(104735-196617) | 164733(122340-212528) | | 160481(117663-205670) | | 134940(100997-172703) |
| Nigeria | 471603  (299655-695649) | | | | | | | | | | | | | | | | | 751780  (461143-1148988) | 1017220  (656641-1451140) | | 1202295  (739845-1763816) | | 1187510  (771230-1707821) |
| North Macedonia | | | | | | | | | | | 3943(2446-5997) | | | | | | | 7143(4274-10962) | 12606(8069-18202) | | 19753(12069-29052) | | 27733(18078-40816) |
| Pakistan | 510385  (329336-748232) | | | | | | | | | | | | | | | | | 788512  (492012-1181942) | 980125  (643103-1376462) | | 1104551  (721796-1498791) | | 1129485  (775030-1510775) |
| Panama | 6132(3679-9267) | | | | | | | | | | | | | | | | | 10821(6298-16855) | 17044(10815-24934) | | 23234(14009-34592) | | 27532(17616-39922) |
| Papua New Guinea | | | | | | | | | | | | | | | 11597(7012-17793) | | | 18258(10733-28509) | 26300(16587-38350) | | 33263(19311-49895) | | 36704(22951-53439) |
| Paraguay | 7855(4662-12256) | | | | | | | | | | | | | | | | | 12191(7037-19261) | 19040(11639-27211) | | 27281(16315-40838) | | 33026(20737-47638) |
| Peru | 46020(27763-70706) | | | | | | | | | | | | | | | | | 74425(43467-117785) | 113931(71185-166818) | | 153193(90535-232608) | | 182213(115481-270021) |
| Philippines | 88044(53250-136914) | | | | | | | | | | | | | | | | | 145854(85006-224519) | 232896(143280-335210) | | 326135(189531-487156) | | 421318(262580-613217) |
| Romania | 37327(22798-57440) | | | | | | | | | | | | | | | | | 67445(38935-105371) | 113830(73624-166681) | | 145721(89654-217688) | | 277923(179672-403366) |
| Russian Federation | 325144  (204333-491991) | | | | | | | | | | | | | | | | | 583601  (352390-891289) | 949124  (615240-1357620) | | 1887389  (1151147-2787392) | | 2691605  (1739267-3892236) |
| Rwanda | 29482(18623-43689) | | | | | | | | | | | | | | | | | 40922(24323-62397) | 56073(35804-81343) | | 74539(44850-112526) | | 83559(53074-124468) |
| Samoa | 220(132-330) | | | | | | | | | | | | | | | | | 410(238-627) | 650(416-949) | | 927(554-1394) | | 1079(683-1581) |
| São Tomé and Principe | | | | | | | | | | | | | | | | | 578(370-854) | 852(513-1291) | 1013(642-1475) | | 1173(713-1746) | | 1132(711-1667) |
| Senegal | 36842(23615-55545) | | | | | | | | | | | | | | | | | 54341(33071-84642) | 70121(43532-103177) | | 85042(52299-125703) | | 87001(54978-124677) |
| Serbia | 15002(9077-23057) | | | | | | | | | | | | | | | | | 26266(15265-40455) | 44634(27933-64061) | | 71096(42505-105780) | | 120822(77656-177030) |
| Sierra Leone | | | | | | | 19561(12411-28934) | | | | | | | | | | | 27901(16631-42780) | 33750(21392-49084) | | 38007(22947-55269) | | 38539(24296-55835) |
| Solomon Islands | | | | | | | | | | | 558(334-860) | | | | | | | 902(520-1398) | 1254(782-1829) | | 1689(1000-2505) | | 2069(1316-3010) |
| Somalia | 45043(28511-65846) | | | | | | | | | | | | | | | | | 57981(35221-89199) | 54597(34804-78823) | | 74112(45537-108922) | | 98193(64040-140614) |
| South Africa | 403416  (275318-572077) | | | | | | | | | | | | | | | | | 614663  (394082-905301) | 760682  (511104-1071576) | | 887530  (568933-1245678) | | 924167  (627389-1274083) |
| South Sudan | | | | | 21035(13219-31538) | | | | | | | | | | | | | 33073(19804-51436) | 43443(27538-63056) | | 4805(29831-71168) | | 47650(30730-70529) |
| Sri Lanka | 34322(20991-53380) | | | | | | | | | | | | | | | | | 58652(34331-92751) | 98631(61696-144702) | | 149709(90071-227536) | | 192642(123105-281923) |
| St. Lucia | 330(199-505) | | | | | | | | | | | | | | | | | 608(350-941) | 963(597-1395) | | 1239(742-1834) | | 1363(861-2018) |
| St. Vincent and the Grenadines | | | | | | | | | | | | 193(121-294) | | | | | | 341(202-533) | 553(345-804) | | 802(475-1196) | | 972(614-1446) |
| Sudan | 26542(16185-40670) | | | | | | | | | | | | | | | | | 39656(23503-62368) | 55337(34252-80804) | | 65424(38638-97871) | | 76935(47618-114082) |
| Suriname | 898(556-1391) | | | | | | | | | | | | | | | | | 1669(994-2540) | 2769(1740-4079) | | 3704(2205-5504) | | 3980(2508-5884) |
| Syrian Arab Republic | 12647(7529-19489) | | | | | | | | | | | | | | | | | 22230(12789-34720) | 36033(22429-52514) | | 48810(29556-72675) | | 55767(35775-80341) |
| Tajikistan | 12556(7595-19501) | | | | | | | | | | | | | | | | | 21214(12381-33569) | 34869(22081-50723) | | 51194(31322-76957) | | 54066(35161-79557) |
| Tanzania | 313550(197738-499055) | | | | | | | | | | | | | | | | | 507416(315355-786079) | 571359(393561-774760) | | 548136(391914-702765) | | 488655(365900-626940) |
| Thailand | 127731(79979-196277) | | | | | | | | | | | | | | | | | 244659(143700-381379) | 423105(258738-614912) | | 619094(359973-917822) | | 766214(488215-1106165) |
| Timor-Leste | 1365(829-2067) | | | | | | | | | | | | | | | | | 2407(1426-3772) | 3155(1977-4645) | | 3988(2286-6001) | | 6417(3974-9434) |
| Togo | 20332(13040-30281) | | | | | | | | | | | | | | | | | 30348(18647-46033) | 37262(23630-53502) | | 41719(24813-61077) | | 39280(25017-56767) |
| Tonga | 115(71-176) | | | | | | | | | | | | | | | | | 203(119-312) | 326(205-470) | | 425(264-629) | | 508(324-741) |
| Tunisia | 11582(6890-18419) | | | | | | | | | | | | | | | | | 17769(10307-28360) | 29353(18433-43345) | | 44109(25464-66599) | | 55913(34261-82638) |
| Turkey | 82279(49939-128986) | | | | | | | | | | | | | | | | | 124308(71903-195630) | 198959(121725-291097) | | 288062(166823-432455) | | 359398(228389-523772) |
| Turkmenistan | | | | | 7287(4470-11105) | | | | | | | | | | | | | 12457(7308-19276) | 20257(12962-28899) | | 31328(19050-46964) | | 35632(22656-51637) |
| Uganda | 71759(43878-110651) | | | | | | | | | | | | | | | | | 107411(63504-166360) | 138379(87919-200820) | | 166116(100328-243277) | | 168236(108797-244519) |
| Ukraine | 68448(41452-106881) | | | | | | | | | | | | | | | | | 124214(72111-191201) | 214715(133697-309352) | | 405215(239389-606973) | | 601563(382886-879615) |
| Uzbekistan | 47487(29318-71629) | | | | | | | | | | | | | | | | | 78121(46259-119812) | 127137(80952-183094) | | 195890(117792-289987) | | 217095(139280-318956) |
| Vanuatu | 291(177-451) | | | | | | | | | | | | | | | | | 469(274-716) | 692(429-1019) | | 947(558-1420) | | 1283(805-1872) |
| Vietnam | 158561(95327-244805) | | | | | | | | | | | | | | | | | 277521(161373-443993) | 439743(283540-642515) | | 642121(396212-961342) | | 745993(475987-1101972) |
| Yemen, Rep. | | | | | 20380(12116-31602) | | | | | | | | | | | | | 26287(15071-40776) | 35741(21617-52239) | | 48055(28294-72364) | | 60653(37821-89427) |
| Zambia | 32533(20516-49412) | | | | | | | | | | | | | | | | | 47187(28296-72024) | 58442(36890-85094) | | 67450(39092-100399) | | 71785(45078-105303) |
| Zimbabwe | 70011(44653-103864) | | | | | | | | | | | | | | | | | 95432(57941-148309) | 105672(67328-154633) | | 120493(72457-174680) | | 135696(87956-194674) |
| Captions: Location according to the GBD 2019 study of Low-and Middle-Income Countries. The number of presbyopia is determined by cases and mortality,  which are according to the GBD 2019 study. The number of cases of presbyopia are given inform with 95% confidence interval. | | | | | | | | | | | | | | | | | | | | | | | |

**Table S5: Work productivity gaps due to vision loss (presbyopia) identified in the literature.**

| **Reference** | **Location and occupation** | **GBD Region** | **VI definition** | **Remarks** | **Relative increase in work productivity.** |
| --- | --- | --- | --- | --- | --- |
| **Naidoo et al^1^** | **Clothing factories in KwaZulu-Natal,**  **Machinists** | **South Africa** | **Near visual acuity (VA) can be improved to at least 6/9 equivalent at near and who were not currently wearing near vision spectacles.** | **This study reported a cross-sectional study among 268 South African textile factory workers aged 40 years and above engaged in various visually intensive tasks in seven clothing factories. Work productivity was calculated as the weight of product (in kilograms) passing quality assurance per day. They included workers who were employed for at least 3 months whose uncorrected near visual acuity could be improved and corrected to better than 6/9 with spectacle correction. Workers were provided with near vision spectacles, and changes in their work productivity were evaluated after 6 months, using the factories’ output records as an indicator for measurement.** | **6.6%** |
|  | **clothing factories in KwaZulu-Natal,**  **quality controllers** |  |  |  | **5.8%** |
| **Pradhan^2^** | **Cotton spinning and textile factory in Madurai** | **India** | **Near visual acuity (VA) can be improved wearing by spectacles.** | **This study reported a longitudinal study compared the impact of presbyopic correction on work productivity among a group of 238 workers engaged in spinning and winding function at a cotton spinning and textile factory. Productivity data was collected for 30 working shifts for each employee. Then the eye test was done by a team comprising of Ophthalmologist and Optometrist from Aravind Eye Hospital (WHO Collaborating Centre) and given the corrective glass to those who were prescribed the correction. After a month of gap given to get used to the glasses, the productivity data was collected again for 30 working shifts for all the samples in the first phase and analyzed.** | **9.5%** |
| **Reddy et al^3^** | **tea pickers in Assam** | **India** | **Unaided near visual acuity (NVA) lower than 6/12 in both eyes, correctable to 6/7·5 with near glasses; unaided distance vision 6/7·5 or greater.** | **This study reported a randomized trial: Participants were randomly assigned (1:1) to receive free glasses optimizing NVA at working distance (cost including delivery US$10·20 per person), intervention group or control group. Participants were stratified by age, sex, and productivity. The primary outcome was the difference between groups in the change in mean daily weight of tea picked (productivity), between the 4-week baseline period (June, 2017) and the 11-week evaluation period (July 24, 2017, to Oct 7, 2017). Workers' income was tied to their productivity.** | **21.7%** |

**Reference:**

**[1]Naidoo KS, Jaggernath J, Chinanayi FS, et al. Near vision correction and work productivity among textile workers. African Vis Eye Heal. 2016;**

**75: a357.**

**[2] Pradhan KB. Impact of uncorrected vision on productivity--a study in an industrial setting a pair of spectacles. J Multidiscip Res Healthc. 2015;1:**

**119–131.**

**[3] Reddy PA, Congdon N, MacKenzie G, et al. Effect of providing near glasses on productivity among rural Indian tea workers with presbyopia**

**(PROSPER): a randomised trial. Lancet Glob Health. 2018;6:e1019–e1027.**

**Table S6. Productivity loss of Presbyopia in 2019 by age group in Millions$.**

| **Location** | **40-44Y** | **45-49Y** | | **50-54Y** | | | **55-59Y** | | **60-64Y** | **40-64Y** |
| --- | --- | --- | --- | --- | --- | --- | --- | --- | --- | --- |
| Afghanistan | 0.270(0.158-0.416) | 0.592(0.337-0.915) | | 0.698(0.428-1.024) | | | 0.505(0.299-0.765) | | 0.393(0.251-0.581) | 2.457(1.471-3.702) |
| Burkina Faso | 2.559(1.628-3.796) | 3.538(2.166-5.355) | | 4.141(2.655-5.955) | | | 4.405(2.769-6.450) | | 3.906(2.532-5.598) | 18.549(11.750-27.154) |
| Burundi | 0.499(0.316-0.754) | 0.680(0.409-1.023) | | 0.880(0.558-1.264) | | | 1.037(0.642-1.512) | | 0.992(0.638-1.457) | 4.087(2.562-6.011) |
| Central African Republic | 0.310(0.193-0.464) | 0.457(0.280-0.698) | | 0.585(0.359-0.845) | | | 0.634(0.369-0.953) | | 0.587(0.362-0.865) | 2.573(1.563-3.825) |
| Chad | 1.573(0.989-2.309) | 2.232(1.352-3.501) | | 2.538(1.600-3.769) | | | 2.623(1.636-3.783) | | 2.297(1.475-3.309) | 11.263(7.053-16.752) |
| Congo, Dem. Rep | 4.416(2.739-6.722) | 6.508(3.846-10.025) | | 8.590(5.434-12.633) | | | 10.006(6.072-15.04) | | 9.467(6.109-13.905) | 38.987(24.199-58.325) |
| Eritrea | 2.161(1.417-3.221) | 3.142(1.945-4.775) | | 3.551(2.293-5.114) | | | 3.562(2.207-5.152) | | 3.041(1.902-4.393) | 15.456(9.764-22.655) |
| Ethiopia | 8.780(5.458-13.296) | 11.825(7.069-17.997) | | 14.977(9.623-21.512) | | | 17.579(10.780-25.891) | | 18.896(12.091-27.194) | 72.058(45.021-105.891) |
| Gambia, The | 0.175(0.111-0.263) | 0.231(0.138-0.360) | | 0.261(0.166-0.383) | | | 0.269(0.165-0.393) | | 0.253(0.161-0.366) | 1.188(0.742-1.765) |
| Guinea | 1.443(0.918-2.17) | 2.056(1.248-3.116) | | 2.395(1.531-3.477) | | | 2.609(1.585-3.833) | | 2.490(1.593-3.670) | 10.993(6.875-16.265) |
| Guinea-Bissau | 0.225(0.143-0.338) | 0.298(0.178-0.453) | | 0.339(0.218-0.491) | | | 0.352(0.219-0.524) | | 0.312(0.201-0.457) | 1.526(0.960-2.263) |
| Korea, Dem. People's Rep | 10.184  (6.353-15.431) | 22.244  (13.34-34.663) | | 35.315  (22.277-52.264) | | | 38.940  (23.634-58.798) | | 39.685  (24.724-58.059) | 146.369  (90.327-219.215) |
| Liberia | 0.721(0.459-1.080) | 1.061(0.644-1.636) | | 0.966(0.615-1.395) | | | 0.983(0.592-1.443) | | 0.831(0.523-1.202) | 4.563(2.833-6.756) |
| Madagascar | 3.129(1.918-4.635) | 4.575(2.712-6.996) | | 5.683(3.607-8.228) | | | 6.338(3.900-9.376) | | 5.782(3.735-8.365) | 25.508(15.873-37.600) |
| Malawi | 1.707(1.096-2.552) | 2.366(1.420-3.546) | | 2.629(1.688-3.782) | | | 2.900(1.771-4.330) | | 3.074(1.969-4.532) | 12.676(7.944-18.742) |
| Mali | 2.566(1.634-3.797) | 3.692(2.249-5.566) | | 4.448(2.833-6.439) | | | 4.823(2.936-7.098) | | 4.430(2.852-6.467) | 19.959(12.504-29.367) |
| Mozambique | 2.299(1.441-3.425) | 3.375(2.054-5.222) | | 4.003(2.542-5.829) | | | 4.399(2.697-6.498) | | 4.301(2.765-6.239) | 18.376(11.500-27.213) |
| Niger | 4.239(3.155-5.682) | 5.754(4.125-7.744) | | 6.171(4.583-7.961) | | | 5.589(4.098-7.163) | | 4.194(3.139-5.368) | 25.947(19.100-33.918) |
| Rwanda | 2.218(1.401-3.287) | 2.987(1.776-4.555) | | 3.911(2.497-5.673) | | | 4.872(2.932-7.355) | | 4.945(3.141-7.367) | 18.934(11.747-28.237) |
| Sierra Leone | 0.474(0.301-0.701) | 0.650(0.387-0.996) | | 0.742(0.470-1.079) | | | 0.771(0.465-1.121) | | 0.691(0.436-1.001) | 3.328(2.059-4.899) |
| Somalia | 0.633(0.400-0.925) | 0.767(0.466-1.179) | | 0.662(0.422-0.955) | | | 0.795(0.488-1.168) | | 0.885(0.577-1.267) | 3.740(2.353-5.494) |
| South Sudan | 2.102(1.321-3.152) | 3.187(1.909-4.957) | | 3.982(2.524-5.779) | | | 4.106(2.549-6.081) | | 3.659(2.360-5.416) | 17.036(10.662-25.385) |
| Sudan | 0.405(0.247-0.620) | 0.591(0.350-0.929) | | 0.796(0.493-1.162) | | | 0.891(0.526-1.334) | | 0.960(0.594-1.423) | 3.643(2.210-5.468) |
| Syrian Arab Republic | 0.378(0.225-0.582) | 0.653(0.375-1.019) | | 1.027(0.639-1.497) | | | 1.327(0.803-1.976) | | 1.397(0.896-2.012) | 4.781(2.939-7.086) |
| Togo | 0.866(0.556-1.29) | 1.240(0.762-1.881) | | 1.435(0.910-2.061) | | | 1.481(0.881-2.168) | | 1.237(0.788-1.787) | 6.259(3.896-9.188) |
| Uganda | 3.745(2.290-5.775) | 5.403(3.194-8.368) | | 6.601(4.194-9.58) | | | 7.366(4.449-10.788) | | 6.693(4.328-9.727) | 29.808(18.455-44.238) |
| Yemen, Rep. | 0.226(0.135-0.351) | 0.284(0.163-0.440) | | 0.369(0.223-0.539) | | | 0.465(0.274-0.700) | | 0.528(0.329-0.779) | 1.872(1.124-2.809) |
| Algeria | 2.787(1.660-4.300) | 4.303(2.452-6.629) | | 6.381(3.946-9.297) | | | 8.159(4.736-12.365) | | 9.364(5.940-13.855) | 30.994(18.734-46.446) |
| Angola | 10.411(6.511-15.653) | 14.367(8.658-21.928) | | 18.895  (12.043-27.639) | | | 22.044  (13.143-33.571) | | 19.903  (12.829-29.579) | 85.621  (53.185-128.37) |
| Bangladesh | 59.740  (38.058-88.710) | 89.727  (53.295-139.114) | | 108.338  (67.894-159.747) | | | 118.127  (71.225-173.347) | | 111.908  (70.388-165.912) | 487.840  (300.860-726.83) |
| Belize | 0.151(0.093-0.228) | 0.236(0.141-0.363) | | 0.314(0.201-0.455) | | | 0.383(0.230-0.564) | | 0.380(0.237-0.554) | 1.465(0.901-2.164) |
| Benin | 2.368(1.501-3.509) | 3.280(2.031-4.969) | | 3.693(2.348-5.380) | | | 3.800(2.339-5.580) | | 3.388(2.176-4.920) | 16.529(10.396-24.358) |
| Bhutan | 0.701(0.446-1.048) | 0.992(0.601-1.518) | | 1.171(0.735-1.711) | | | 1.262(0.746-1.862) | | 1.186(0.734-1.743) | 5.312(3.262-7.882) |
| Bolivia | 2.853(1.757-4.378) | 4.303(2.524-6.712) | | 6.174(3.906-9.035) | | | 8.206(4.795-12.426) | | 9.587(6.050-14.033) | 31.123(19.033-46.584) |
| Cabo Verde | 0.240(0.150-0.355) | 0.363(0.215-0.567) | | 0.502(0.316-0.732) | | | 0.592(0.366-0.858) | | 0.570(0.365-0.832) | 2.268(1.412-3.344) |
| Cambodia | 3.056(1.858-4.687) | 4.832(2.901-7.428) | | 8.019(5.160-11.797) | | | 10.602(6.487-16.155) | | 11.222(7.228-16.542) | 37.731(23.634-56.609) |
| Cameroon | 7.698(4.946-11.716) | 10.463(6.258-15.952) | | 12.107(7.724-17.53) | | | 12.990(7.894-19.048) | | 11.575(7.456-16.789) | 54.834(34.277-81.035) |
| Comoros | 0.071(0.045-0.106) | 0.112(0.067-0.172) | | 0.143(0.091-0.205) | | | 0.160(0.097-0.237) | | 0.156(0.101-0.228) | 0.642(0.400-0.948) |
| Congo, Rep. | 1.370(0.832-2.098) | 1.996(1.159-3.104) | | 2.408(1.524-3.460) | | | 2.611(1.592-3.901) | | 2.409(1.547-3.509) | 10.793(6.654-16.072) |
| Côte d'Ivoire | 6.995(4.426-10.640) | 9.326(5.539-14.495) | | 10.454(6.485-15.250) | | | 10.897(6.617-16.167) | | 9.359(5.985-13.892) | 47.032(29.052-70.443) |
| Djibouti | 0.535(0.333-0.803) | 0.751(0.446-1.156) | | 0.887(0.568-1.294) | | | 0.937(0.562-1.392) | | 0.858(0.537-1.239) | 3.967(2.446-5.883) |
| Egypt, Arab Rep. | 4.909(2.877-7.551) | 7.222(4.124-11.182) | | 11.015(6.699-16.168) | | | 14.558(8.263-22.085) | | 15.925(9.744-23.696) | 53.628(31.707-80.681) |
| El Salvador | 1.375(0.856-2.099) | 2.211(1.321-3.414) | | 3.335(2.088-4.867) | | | 4.414(2.712-6.691) | | 5.157(3.289-7.584) | 16.492(10.266-24.655) |
| Eswatini | 0.671(0.432-0.993) | 0.865(0.522-1.321) | | 0.950(0.602-1.375) | | | 0.977(0.598-1.453) | | 0.916(0.591-1.346) | 4.379(2.744-6.488) |
| Ghana | 9.549  (5.906-14.65) | 13.574  (8.194-20.856) | | 16.162  (10.422-23.200) | | | 17.639  (11.089-25.504) | | 17.065  (11.137-24.052) | 73.990  (46.748-108.263) |
| Haiti | 1.238(0.746-1.861) | 1.706(1.000-2.591) | | 2.185(1.388-3.146) | | | 2.615(1.587-3.858) | | 2.701(1.697-3.961) | 10.446(6.418-15.417) |
| Honduras | 1.589(0.982-2.436) | 2.382(1.379-3.674) | | 3.260(2.017-4.740) | | | 4.200(2.482-6.309) | | 4.698(3.035-7.008) | 16.129(9.896-24.167) |
| India | 452.130  (293.644-665.238) | 674.317  (417.682-1022.487) | | 796.667  (513.304-1156.300) | | | 854.497  (522.422-1237.232) | | 834.458  (539.067-1206.107) | 3612.068  (2286.118-5287.363) |
| Indonesia | 61.727  (37.439-94.978) | 100.147  (58.429-155.128) | | 152.018  (94.536-221.869) | | | 199.450  (119.439-299.011) | | 223.513  (140.520-326.264) | 736.855  (450.363-1097.250) |
| Iran, Islamic Rep | 3.631(2.131-5.700) | 5.360(3.049-8.361) | | 8.129(4.854-11.937) | | | 10.474(5.964-15.889) | | 13.306(8.156-19.330) | 40.899(24.152-61.216) |
| Kenya | 8.972  (5.639-13.652) | 13.109  (7.885-20.141) | | 17.468  (11.157-25.089) | | | 21.332  (13.083-31.732) | | 22.064  (14.225-32.043) | 82.945  (51.988-122.658) |
| Kiribati | 0.004(0.002-0.006) | 0.005(0.003-0.008) | | 0.008(0.005-0.012) | | | 0.011(0.007-0.017) | | 0.012(0.007-0.017) | 0.041(0.025-0.061) |
| Kyrgyz Republic | 0.511(0.313-0.754) | 0.872(0.518-1.338) | | 1.359(0.841-1.998) | | | 1.949(1.161-2.965) | | 2.008(1.272-2.942) | 6.698(4.105-9.997) |
| Lao PDR | 2.001(1.223-3.096) | 3.120(1.830-4.775) | | 4.476(2.778-6.563) | | | 5.657(3.298-8.410) | | 5.803(3.691-8.512) | 21.056(12.819-31.355) |
| Lesotho | 0.621(0.408-0.912) | 0.769(0.475-1.170) | | 0.944(0.610-1.370) | | | 1.055(0.663-1.534) | | 0.962(0.628-1.379) | 4.351(2.784-6.364) |
| Mauritania | 0.410(0.258-0.608) | 0.601(0.364-0.901) | | 0.727(0.451-1.040) | | | 0.810(0.482-1.179) | | 0.778(0.485-1.149) | 3.327(2.039-4.877) |
| Mongolia | 1.333(0.818-2.045) | 2.130(1.263-3.301) | | 3.170(2.002-4.543) | | | 4.072(2.420-6.115) | | 3.727(2.391-5.418) | 14.431(8.894-21.421) |
| Morocco | 2.385(1.399-3.849) | 3.794(2.140-6.042) | | 5.860(3.570-8.776) | | | 8.355(4.828-12.681) | | 9.592(5.988-14.058) | 29.986(17.924-45.406) |
| Myanmar | 4.652(2.879-7.077) | 7.729(4.524-12.198) | | 11.744(7.338-16.989) | | | 15.499(9.499-23.033) | | 16.908(10.815-24.27) | 56.532(35.054-83.567) |
| Nepal | 31.833  (23.365-40.517) | 52.020  (36.713-69.975) | | 58.012  (43.605-74.059) | | | 54.253  (39.720-67.596) | | 44.664  (32.730-55.426) | 240.782  (176.133-307.573) |
| Nicaragua | 0.932(0.581-1.485) | 1.421(0.844-2.265) | | 2.069(1.303-3.000) | | | 2.678(1.596-3.973) | | 2.943(1.880-4.287) | 10.043(6.204-15.010) |
| Nigeria | 38.626  (24.543-56.976) | 59.616  (36.568-91.114) | | 77.165  (49.812-110.081) | | | 85.742  (52.762-125.787) | | 77.026  (50.025-110.776) | 338.174  (213.710-494.734) |
| Pakistan | 23.728  (15.311-34.785) | 35.461  (22.127-53.155) | | 41.896  (27.490-58.837) | | | 43.818  (28.634-59.458) | | 39.902  (27.380-53.372) | 184.805  (120.941-259.607) |
| Papua New Guinea | 1.063(0.642-1.63) | 1.600(0.941-2.499) | | 2.163(1.364-3.154) | | | 2.498(1.450-3.747) | | 2.409(1.506-3.507) | 9.732(5.904-14.537) |
| Philippines | 13.690  (8.28-21.289) | 22.086  (12.872-33.998) | | 33.911  (20.863-48.809) | | | 44.758  (26.011-66.857) | | 52.925  (32.985-77.031) | 167.371  (101.010-247.984) |
| Samoa | 0.023(0.014-0.035) | 0.042(0.024-0.064) | | 0.063(0.040-0.092) | | | 0.083(0.050-0.125) | | 0.087(0.055-0.128) | 0.298(0.183-0.443) |
| São Tomé and Principe | 0.043(0.028-0.064) | 0.062(0.037-0.094) | | 0.071(0.045-0.103) | | | 0.077(0.047-0.115) | | 0.068(0.042-0.100) | 0.321(0.199-0.476) |
| Senegal | 1.374(0.881-2.072) | 1.969(1.199-3.068) | | 2.434(1.511-3.581) | | | 2.770(1.703-4.094) | | 2.563(1.619-3.672) | 11.11(6.913-16.487) |
| Solomon Islands | 0.188(0.112-0.289) | 0.277(0.160-0.429) | | 0.339(0.211-0.494) | | | 0.382(0.226-0.566) | | 0.368(0.234-0.536) | 1.554(0.944-2.314) |
| Sri Lanka | 4.165(2.547-6.478) | 7.009(4.103-11.084) | | 11.517(7.204-16.897) | 16.914(10.176-25.707) | | | 20.660(13.203-30.235) | | 60.266(37.233-90.402) |
| Tajikistan | 0.215(0.130-0.334) | 0.356(0.208-0.564) | | 0.567(0.359-0.825) | | | 0.786(0.481-1.181) | | 0.750(0.488-1.103) | 2.674(1.665-4.007) |
| Tanzania | 32.322  (20.384-51.445) | 50.562  (31.424-78.330) | | 54.267  (37.380-73.586) | | | 48.720  (34.834-62.463) | | 39.260  (29.397-50.370) | 225.131  (153.419-316.194) |
| Timor-Leste | 0.126(0.076-0.191) | 0.217(0.128-0.340) | | 0.273(0.171-0.403) | | | 0.326(0.187-0.409) | | 0.476(0.295-0.700) | 1.418(0.858-2.123) |
| Tunisia | 0.899(0.535-1.429) | 1.362(0.79-2.175) | | 2.209(1.387-3.263) | | | 3.216(1.857-4.856) | | 3.870(2.371-5.720) | 11.557(6.940-17.442) |
| Ukraine | 8.589  (5.202-13.412) | 15.157  (8.799-23.331) | | 25.214  (15.700-36.327) | | | 45.088  (26.637-67.538) | | 61.790  (39.328-90.350) | 155.838  (95.666-230.958) |
| Uzbekistan | 4.165(2.571-6.283) | 6.671(3.950-10.231) | | 10.394(6.618-14.968) | | | 14.923(8.974-22.092) | | 14.531(9.322-21.348) | 50.684(31.436-74.922) |
| Vanuatu | 0.067(0.041-0.105) | 0.103(0.060-0.158) | | 0.142(0.088-0.209) | | | 0.175(0.103-0.262) | | 0.205(0.128-0.299) | 0.692(0.421-1.032) |
| Vietnam | 32.067  (19.279-49.509) | 54.985  (31.973-87.968) | | 84.351  (54.388-123.246) | | | 117.259  (72.353-175.552) | | 126.305  (80.590-186.576) | 414.966  (258.582-622.850) |
| Zambia | 3.270(2.062-4.967) | 4.479(2.686-6.836) | | 5.131(3.239-7.471) | | | 5.352(3.102-7.967) | | 4.958(3.113-7.272) | 23.190(14.202-34.514) |
| Zimbabwe | 9.937  (6.338-14.743) | 12.616  (7.660-19.606) | | 12.689  (8.085-18.568) | | | 12.842  (7.723-18.618) | | 12.345  (8.002-17.711) | 60.430  (37.807-89.245) |
| Albania | 0.885(0.541-1.375) | 1.811(1.093-2.828) | | 3.434(2.167-4.981) | | | 5.705(3.272-8.534) | | 7.280(4.484-10.643) | 19.114(11.557-28.361) |
| Argentina | 3.035(1.629-5.013) | 4.300(2.229-7.045) | | 6.007(3.313-9.290) | | | 8.431(4.343-13.425) | | 11.03(6.303-17.491) | 32.803(17.818-52.264) |
| Armenia | 0.620(0.380-0.944) | 0.992(0.576-1.542) | | 1.741(1.087-2.528) | | | 3.325(1.982-5.112) | | 4.399(2.734-6.483) | 11.077(6.759-16.609) |
| Azerbaijan | 4.111  (2.524-6.242) | 7.249  (4.272-11.306) | | 13.197  (8.252-19.128) | | | 20.416  (12.315-30.219) | | 20.598  (13.317-29.843) | 65.571  (40.681-96.738) |
| Belarus | 7.736  (4.831-11.546) | 13.912  (8.308-21.071) | | 23.419  (14.755-34.024) | | | 41.313  (24.717-62.736) | | 51.901  (33.584-76.112) | 138.282  (86.195-205.49) |
| Bosnia and Herzegovina | 0.899(0.537-1.376) | 1.564(0.924-2.422) | | 2.841(1.786-4.122) | | | 4.696(2.708-6.992) | | 6.298(3.929-9.290) | 16.297(9.885-24.201) |
| Botswana | 6.389(4.103-9.497) | 8.185(5.093-12.679) | | 8.623(5.507-12.578) | | | 9.295(5.660-13.556) | | 8.310(5.311-11.995) | 40.802(25.673-60.304) |
| Brazil | 70.166  (42.332-107.974) | 110.857  (64.600-173.496) | | 183.192  (111.960-265.237) | | | 261.512  (152.750-393.414) | | 323.304  (201.509-466.027) | 949.032  (573.150-1406.149) |
| Bulgaria | 4.861  (2.932-7.476) | 8.734  (5.149-13.424) | | 13.900  (8.713-20.368) | | | 21.491  (12.799-31.743) | | 30.385  (19.336-43.837) | 79.371  (48.929-116.848) |
| China | 2398.741  (1553.870  -3550.713) | 5415.447  (3367.438  -8253.252) | | 8933.509  (5823.876  -12758.356) | | | 9919.642  (6150.926  -14440.356) | | 10343.406  (6732.225  -14861.196) | 37010.746  (23628.335  -53863.873) |
| Colombia | 20.374  (12.187-30.739) | 35.217  (20.739-54.314) | | 60.113  (37.405-86.938) | | | 84.633  (49.726-126.993) | | 97.043  (61.784-141.063) | 297.379  (181.841-440.047) |
| Costa Rica | 4.354  (2.615-6.634) | 7.081  (4.194-10.799) | | 12.192  (7.727-17.604) | | | 17.615  (10.690-26.452) | | 20.265  (13.210-29.834) | 61.506  (38.437-91.323) |
| Cuba | 5.444  (3.306-8.230) | 13.341  (7.673-20.494) | | 22.454  (14.372-32.516) | | | 28.897  (17.859-43.028) | | 25.992  (16.939-37.927) | 96.128  (60.151-142.195) |
| Dominican Republic | 7.154  (4.436-10.878) | 11.281  (6.671-17.345) | | 15.746  (9.935-23.084) | | | 19.288  (11.611-29.284) | | 20.066  (12.736-29.431) | 73.535  (45.389-110.022) |
| Ecuador | 6.380(3.800-9.731) | 10.109(5.980-15.767) | 14.980(9.081-21.858) | | 20.604(12.023-31.056) | | | | 23.120(14.206-33.217) | 75.192(45.089-111.629) |
| Equatorial Guinea | 0.743(0.472-1.107) | 1.022(0.608-1.600) | | 1.265(0.778-1.865) | | | 1.391(0.806-2.113) | | 1.357(0.851-1.996) | 5.779(3.515-8.681) |
| Fiji | 0.309(0.187-0.477) | 0.489(0.288-0.755) | | 0.806(0.493-1.187) | | | 1.123(0.652-1.715) | | 1.152(0.723-1.697) | 3.879(2.343-5.832) |
| Gabon | 0.779(0.483-1.197) | 1.202(0.719-1.839) | | 1.623(1.020-2.329) | | | 1.951(1.175-2.885) | | 1.887(1.202-2.730) | 7.442(4.599-10.980) |
| Georgia | 1.294(0.772-2.047) | 2.310(1.370-3.646) | | 3.954(2.437-5.822) | | | 6.896(3.979-10.383) | | 9.014(5.613-13.069) | 23.468(14.171-34.967) |
| Guatemala | 4.570(2.838-7.283) | 6.429(3.817-10.272) | | 8.850(5.574-12.842) | | | 11.704(6.98-17.373) | | 13.59(8.685-19.795) | 45.143(27.895-67.566) |
| Guyana | 0.245(0.150-0.379) | 0.396(0.228-0.631) | | 0.571(0.354-0.847) | | | 0.728(0.426-1.097) | | 0.744(0.466-1.104) | 2.684(1.624-4.059) |
| Iraq | 3.466(2.080-5.360) | 5.633(3.238-8.785) | | 7.160(4.443-10.634) | | | 8.457(5.082-12.823) | | 9.260(5.912-13.527) | 33.976(20.756-51.129) |
| Jamaica | 1.151(0.708-1.779) | 2.020(1.194-3.113) | | 3.007(1.907-4.381) | | | 3.903(2.382-5.799) | | 4.235(2.766-6.258) | 14.316(8.957-21.331) |
| Jordan | 0.592(0.347-0.921) | 0.948(0.540-1.486) | | 1.353(0.821-1.996) | | | 1.563(0.891-2.430) | | 1.517(0.936-2.246) | 5.974(3.536-9.079) |
| Kazakhstan | 16.421  (9.991-25.015) | 27.633  (16.163-42.508) | | 42.627  (26.288-62.139) | | | 64.769  (38.501-96.065) | | 70.912  (44.824-103.043) | 222.361  (135.767-328.769) |
| Lebanon | 0.781(0.470-1.213) | 1.303(0.752-2.029) | | 1.922(1.206-2.818) | | | 2.700(1.659-4.107) | | 2.970(1.894-4.374) | 9.677(5.980-14.541) |
| Libya | 1.374(0.810-2.138) | 2.064(1.197-3.248) | | 2.678(1.671-3.895) | | | 2.924(1.698-4.391) | | 2.943(1.829-4.283) | 11.983(7.205-17.955) |
| Malaysia | 24.340  (14.483-36.969) | 38.014  (22.156-58.981) | | 58.641  (37.014-85.460) | | | 80.804  (47.006-120.933) | | 93.470  (57.842-136.507) | 295.271  (178.501-438.849) |
| Maldives | 0.526(0.320-0.817) | 0.717(0.410-1.129) | | 0.942(0.574-1.371) | | | 1.149(0.651-1.721) | | 1.149(0.725-1.649) | 4.484(2.681-6.687) |
| Mauritius | 0.896(0.550-1.373) | 1.457(0.851-2.275) | | 2.711(1.674-4.001) | | | 4.138(2.485-6.259) | | 4.827(3.060-7.113) | 14.027(8.621-21.021) |
| Mexico | 62.661  (38.489-96.202) | 107.624  (63.787-166.016) | | 166.333  (103.367-240.467) | | | 223.586  (131.149-334.271) | | 270.090  (169.564-391.734) | 830.294  (506.357-1228.689) |
| Moldova | 0.977(0.58-1.464) | 1.620(0.956-2.481) | | 2.600(1.631-3.774) | | | 4.553(2.73-6.825) | | 5.534(3.477-8.117) | 15.284(9.374-22.662) |
| Montenegro | 0.304(0.184-0.465) | 0.530(0.303-0.827) | | 0.912(0.571-1.345) | | | 1.464(0.903-2.179) | | 1.956(1.247-2.784) | 5.166(3.208-7.601) |
| Namibia | 2.636(1.706-3.888) | 3.691(2.253-5.671) | | 4.117(2.675-6.053) | | | 4.175(2.612-6.132) | | 3.723(2.398-5.349) | 18.342(11.645-27.094) |
| North Macedonia | 0.717(0.445-1.091) | 1.278(0.765-1.961) | | 2.196(1.405-3.170) | | | 3.281(2.005-4.826) | | 4.275(2.787-6.292) | 11.747(7.406-17.339) |
| Panama | 4.406(2.643-6.658) | 7.676(4.468-11.957) | | 11.870(7.532-17.365) | | | 15.741(9.491-23.436) | | 17.908(11.458-25.967) | 57.602(35.591-85.384) |
| Paraguay | 2.531(1.502-3.949) | 3.857(2.226-6.094) | | 5.856(3.580-8.370) | | | 8.051(4.815-12.052) | | 9.14(5.739-13.183) | 29.435(17.862-43.648) |
| Peru | 18.437  (11.123-28.327) | 29.456  (17.203-46.617) | | 44.307  (27.683-64.875) | | | 58.063  (34.314-88.162) | | 66.400  (42.082-98.398) | 216.663  (132.406-326.379) |
| Romania | 18.293  (11.173-28.150) | 32.354  (18.678-50.548) | | 52.692  (34.080-77.156) | | | 63.833  (39.273-95.357) | | 112.842  (72.951-163.775) | 280.015  (176.154-414.987) |
| Russian Federation | 179.414  (112.751-271.480) | 312.217  (188.523-476.825) | | 486.588  (315.415-696.012) | | | 912.930  (556.810-1348.261) | | 1195.156  (772.289-1728.274) | 3086.304  (1945.787-4520.851) |
| Serbia | 3.901(2.360-5.996) | 6.721(3.906-10.351) | | 11.089(6.940-15.915) | | 16.831(10.063-25.042) | | | 26.575(17.081-38.938) | 65.117(40.349-96.242) |
| South Africa | 80.545  (54.969-114.219) | 116.279  (74.551-171.261) | | 134.944  (90.669-190.096) | | | 145.866  (93.504-204.728) | | 137.359  (93.249-189.367) | 614.993  (406.942-869.671) |
| St. Lucia | 0.205(0.124-0.313) | 0.369(0.212-0.571) | | 0.566(0.351-0.820) | | | 0.698(0.418-1.032) | | 0.718(0.453-1.062) | 2.555(1.558-3.799) |
| St. Vincent and the Grenadines | 0.065(0.041-0.099) | 0.112(0.067-0.175) | | 0.175(0.109-0.254) | | | 0.241(0.142-0.359) | | 0.271(0.171-0.403) | 0.865(0.530-1.291) |
| Suriname | 0.187(0.116-0.289) | 0.339(0.202-0.515) | | 0.541(0.34-0.797) | | | 0.684(0.407-1.017) | | 0.678(0.427-1.002) | 2.429(1.492-3.621) |
| Thailand | 53.811  (33.694-82.688) | 101.134  (59.401-157.65) | | 170.591  (104.32-247.925) | | | 241.516  (140.430-358.053) | | 284.744  (181.433-411.078) | 851.795  (519.277-1257.394) |
| Tonga | 0.016(0.010-0.024) | 0.027(0.016-0.041) | | 0.041(0.026-0.060) | | | 0.051(0.032-0.075) | | 0.056(0.035-0.081) | 0.19(0.119-0.281) |
| Turkey | 20.906  (12.689-32.774) | 31.249  (18.075-49.178) | | 49.083  (30.030-71.814) | | | 68.935  (39.922-103.49) | | 81.749  (51.949-119.137) | 251.922  (152.665-376.393) |
| Turkmenistan | 2.415(1.482-3.681) | 4.011(2.353-6.207) | | 6.251(4.000-8.917) | | | 9.046(5.500-13.560) | | 9.251(5.882-13.406) | 30.974(19.217-45.772) |
| TOTAL | 3973.37  (2551.255  -5909.088) | 7905.691  (4875.564  -12063.224) | | 12365.742  (8014.763  -17711.561) | | | 14441.226  (8891.462  -21095.757) | | 15446.336  (10007.082  -22238.219) | 54132.365  (34340.126  -79017.85) |
| Captions：Location according to the GBD 2019 study of Low-and Middle-Income Countries. The productivity loss of presbyopia are given inform with 95% confidence interval. | | | | | | | | | | |

**Table S7. Productivity loss of Presbyopia in 2019 by age group until retirement in Millions$.**

| **Location** | **40-44Y** | **45-49Y** | | **50-54Y** | **55-59Y** | | **60-64Y** | | | **40-64Y** | |
| --- | --- | --- | --- | --- | --- | --- | --- | --- | --- | --- | --- |
| Afghanistan | 2.812(1.643-4.339) | 5.207(2.959-8.046) | | 4.714(2.89-6.918) | 2.036(1.205-3.089) | | 0.393(0.251-0.581) | | | 15.161(8.947-22.972) | |
| Burkina Faso | 28.805  (18.326-42.734) | 33.241  (20.351-50.311) | | 29.480  (18.903-42.391) | 18.306  (11.508-26.807) | | 3.906  (2.532-5.598) | | | 113.738  (71.62-167.841) | |
| Burundi | 5.423(3.434-8.202) | 6.201(3.731-9.336) | | 6.123(3.884-8.797) | 4.256(2.635-6.209) | | 0.992(0.638-1.457) | | | 22.995(14.322-34.001) | |
| Central African Republic | 2.499(1.552-3.74) | 3.27(2.001-4.994) | | 3.418(2.097-4.936) | 2.373(1.383-3.569) | | 0.587(0.362-0.865) | | | 12.147(7.395-18.103) | |
| Chad | 17.283(10.865-26.26) | 20.582(12.468-32.283) | | 17.838(11.245-26.492) | 10.823(6.752-15.61) | | 2.297(1.475-3.309) | | | 68.823(42.806-103.954) | |
| Congo, Dem. Rep | 49.029  (30.411-74.636) | 60.490  (35.749-93.169) | | 60.667  (38.375-89.224) | 41.431  (25.140-62.277) | | 9.467  (6.109-13.905) | | | 221.084  (135.783-333.211) | |
| Eritrea | 22.443(14.719-33.458) | | 27.634(17.108-42.002) | 24.091(15.561-34.696) | 14.441(8.947-20.89) | | 3.041(1.902-4.393) | | | 91.651(58.236-135.439) | |
| Ethiopia | 107.165  (66.615-162.292) | 119.316  (71.32-181.589) | | 112.713  (72.423-161.896) | 75.504  (46.303-111.205) | | 18.896  (12.091-27.194) | | | 433.594  (268.751-644.176) | |
| Gambia, The | 1.965(1.244-2.944) | 2.166(1.3-3.383) | | 1.862(1.187-2.734) | 1.12(0.69-1.639) | | 0.253(0.161-0.366) | | | 7.366(4.581-11.066) | |
| Guinea | 15.746(10.017-23.685) | 18.849(11.445-28.561) | | 16.753(10.706-24.323) | 10.737(6.523-15.773) | | 2.49(1.593-3.67) | | | 64.576(40.285-96.012) | |
| Guinea-Bissau | 2.259(1.438-3.39) | 2.555(1.527-3.88) | | 2.259(1.453-3.27) | 1.41(0.88-2.102) | | 0.312(0.201-0.457) | | | 8.796(5.499-13.099) | |
| Korea, Dem. People's Rep | 124.734  (77.804-188.993) | 224.508  (134.636-349.859) | | 265.342  (167.38-392.69) | 167.243  (101.503-252.527) | | 39.685  (24.724-58.059) | | | 821.512  (506.048-1242.128) | |
| Liberia | 8.388(5.338-12.561) | 10.291(6.244-15.868) | | 7.059(4.494-10.194) | 4.151(2.502-6.091) | | 0.831(0.523-1.202) | | | 30.719(19.1-45.916) | |
| Madagascar | 35.167(21.555-52.085) | 42.93(25.444-65.648) | | 40.389(25.636-58.476) | | 26.325(16.2-38.94) | 5.782(3.735-8.365) | | | 150.593(92.569-223.514) | |
| Malawi | 18.917(12.152-28.285) | 22.086(13.251-33.104) | | 18.746(12.036-26.969) | 12.109(7.396-18.077) | | 3.074(1.969-4.532) | | | 74.931(46.803-110.967) | |
| Mali | 29.889  (19.035-44.23) | 35.810  (21.808-53.986) | | 32.487  (20.695-47.034) | 20.354  (12.39-29.952) | | 4.430  (2.852-6.467) | | | 122.969  (76.78-181.669) | |
| Mozambique | 22.374  (14.027-33.332) | 28.464  (17.326-44.043) | | 26.537  (16.854-38.644) | 17.657  (10.825-26.084) | | 4.301  (2.765-6.239) | | | 99.333  (61.798-148.342) | |
| Niger | 49.212  (36.626-65.959) | 55.512  (39.798-74.712) | | 44.816  (33.283-57.819) | 23.489  (17.222-30.103) | | 4.194  (3.139-5.368) | | | 177.223  (130.069-233.961) | |
| Rwanda | 26.534(16.761-39.321) | 29.608(17.599-45.146) | | 29.047(18.547-42.137) | 20.774(12.499-31.36) | | | 4.945(3.141-7.367) | | | 110.908(68.547-165.331) |
| Sierra Leone | 5.335(3.385-7.892) | 6.118(3.647-9.38) | | 5.298(3.358-7.706) | 3.21(1.938-4.668) | | 0.691(0.436-1.001) | | | 20.653(12.764-30.647) | |
| Somalia | 6.171(3.906-9.021) | 6.386(3.879-9.824) | | 4.305(2.744-6.215) | 3.144(1.932-4.621) | | 0.885(0.577-1.267) | | | 20.89(13.038-30.947) | |
| South Sudan | 24.565  (15.437-36.83) | 31.050  (18.593-48.289) | | 29.201  (18.509-42.383) | 17.376  (10.787-25.736) | | 3.659  (2.36-5.416) | | | 105.850  (65.687-158.655) | |
| Sudan | 5.036(3.071-7.717) | 6.049(3.585-9.513) | | 6.043(3.741-8.825) | 3.844(2.27-5.75) | | 0.96(0.594-1.423) | | | 21.932(13.261-33.229) | |
| Syrian Arab Republic | 4.816(2.867-7.422) | 6.806(3.915-10.63) | | 7.899(4.916-11.511) | 5.756(3.486-8.571) | | 1.397(0.896-2.012) | | | 26.674(16.081-40.146) | |
| Togo | 9.735(6.243-14.498) | 11.681(7.177-17.719) | | 10.269(6.512-14.744) | 6.185(3.679-9.055) | | 1.237(0.788-1.787) | | | 39.107(24.399-57.803) | |
| Uganda | 43.410  (26.544-66.938) | 52.236  (30.883-80.904) | | 48.181  (30.612-69.922) | 31.117  (18.794-45.571) | | 6.693  (4.328-9.727) | | | 181.637  (111.161-273.063) | |
| Yemen, Rep. | 2.7(1.605-4.187) | 2.8(1.605-4.343) | | 2.725(1.648-3.983) | 1.971(1.161-2.969) | | 0.528(0.329-0.779) | | | 10.724(6.349-16.26) | |
| Algeria | 37.675  (22.438-58.138) | 47.406  (27.014-73.03) | | 51.382  (31.775-74.858) | 36.462  (21.166-55.259) | | 9.364  (5.94-13.855) | | | 182.290  (108.333-275.141) | |
| Angola | 116.344  (72.76-174.92) | 134.801  (81.235-205.74) | | 134.780  (85.907-197.148) | 91.845  (54.761-139.873) | | 19.903  (12.829-29.579) | | | 497.674  (307.492-747.26) | |
| Bangladesh | 775.418  (493.98-1151.441) | 951.833  (565.356-1475.727) | | 848.824  (531.947-1251.613) | 519.525  (313.247-762.385) | | 111.908  (70.388-165.912) | | | 3207.507  (1974.919-4807.077) | |
| Belize | 1.912(1.177-2.891) | 2.468(1.473-3.792) | | 2.43(1.553-3.518) | 1.68(1.008-2.471) | | 0.38(0.237-0.554) | | | 8.871(5.448-13.227) | |
| Benin | 27.48(17.423-40.717) | 31.651(19.593-47.942) | | 26.849(17.07-39.111) | 15.986(9.842-23.476) | | | | 3.388(2.176-4.92) | 105.354(66.104-156.166) | |
| Bhutan | 9.089(5.784-13.584) | 10.514(6.373-16.096) | | 9.134(5.734-13.35) | 5.535(3.269-8.163) | | 1.186(0.734-1.743) | | | 35.458(21.895-52.936) | |
| Bolivia | 35.959  (22.151-55.181) | 44.508  (26.11-69.424) | | 47.237  (29.882-69.125) | 35.550  (20.773-53.832) | | 9.587  (6.05-14.033) | | | 172.841  (104.966-261.596) | |
| Cabo Verde | 3.048(1.909-4.514) | 3.788(2.246-5.921) | | 3.88(2.441-5.654) | 2.589(1.599-3.75) | | 0.57(0.365-0.832) | | | 13.875(8.56-20.671) | |
| Cambodia | 36.611  (22.258-56.146) | 47.870  (28.742-73.587) | | 59.434  (38.246-87.43) | 45.139  (27.618-68.783) | | 11.222  (7.228-16.542) | | | 200.278  (124.092-302.488) | |
| Cameroon | 84.983  (54.594-129.33) | 97.456  (58.286-148.579) | | 86.120  (54.941-124.691) | 54.086  (32.869-79.308) | | 11.575  (7.456-16.789) | | | 334.221  (208.146-498.697) | |
| Comoros | 0.854(0.543-1.288) | 1.114(0.668-1.715) | | 1.070(0.68-1.531) | 0.683(0.413-1.01) | | 0.156(0.101-0.228) | | | 3.877(2.405-5.771) | |
| Congo, Rep. | 14.868(9.031-22.772) | 18.276(10.613-28.414) | | 16.86(10.676-24.23) | 10.782(6.575-16.112) | | 2.409(1.547-3.509) | | | 63.195(38.442-95.037) | |
| Côte d'Ivoire | 78.734  (49.823-119.759) | 87.973  (52.248-136.726) | | 74.888  (46.453-109.24) | 45.517  (27.639-67.526) | | 9.359  (5.985-13.892) | | | 296.471  (182.149-447.142) | |
| Djibouti | 6.170(3.846-9.267) | 7.257(4.304-11.161) | | 6.473(4.146-9.443) | 3.958(2.376-5.88) | | 0.858(0.537-1.239) | | | 24.715(15.208-36.99) | |
| Egypt, Arab Rep. | 57.578  (33.752-88.567) | 69.685  (39.794-107.895) | | 79.534  (48.366-116.74) | 60.791  (34.506-92.223) | | 15.925  (9.744-23.696) | | | 283.512  (166.162-429.121) | |
| El Salvador | 17.767(11.063-27.118) | 23.505(14.042-36.303) | | 26.217(16.41-38.259) | 19.516(11.991-29.581) | | 5.157(3.289-7.584) | | | 92.163(56.795-138.844) | |
| Eswatini | 6.114(3.934-9.047) | 6.973(4.208-10.648) | | 6.151(3.898-8.907) | 3.894(2.382-5.789) | | 0.916(0.591-1.346) | | | 24.048(15.014-35.737) | |
| Ghana | 109.323  (67.616-167.721) | 129.905  (78.414-199.589) | | 116.970  (75.426-167.907) | 74.099  (46.585-107.142) | | 17.065  (11.137-24.052) | | | 447.363  (279.179-666.410) | |
| Haiti | 13.699(8.256-20.596) | 15.861(9.295-24.078) | | 15.47(9.823-22.268) | 10.85(6.585-16.008) | | 2.701(1.697-3.961) | | | 58.581(35.655-86.91) | |
| Honduras | 19.333  (11.939-29.631) | 23.885  (13.832-36.842) | | 24.361  (15.075-35.419) | 17.973  (10.622-26.997) | | 4.698  (3.035-7.008) | | | 90.249  (54.503-135.897) | |
| India | 5576.02  (3621.452-8204.238) | 6847.649  (4241.534-10383.297) | | 6022.463  (3880.358-8741.138) | 3685.699  (2253.361-5336.549) | | 834.458  (539.067-1206.107) | | | 22966.288  (14535.771-33871.328) | |
| Indonesia | 758.821  (460.245-1167.573) | 1012.842  (590.927-1568.902) | | 1142.445  (710.453-1667.389) | 855.026  (512.026-1281.839) | | 223.513  (140.52-326.264) | | | 3992.647  (2414.171-6011.966) | |
| Iran, Islamic Rep | 49.360  (28.964-77.487) | 59.264  (33.711-92.448) | | 65.586  (39.161-96.31) | 46.898  (26.705-71.145) | | 13.306  (8.156-19.33) | | | 234.414  (136.696-356.72) | |
| Kenya | 100.965  (63.452-153.634) | 124.311  (74.769-190.996) | | 126.150  (80.574-181.188) | 89.798  (55.073-133.58) | | 22.064  (14.225-32.043) | | | 463.288  (288.094-691.441) | |
| Kiribati | 0.034(0.021-0.053) | 0.042(0.024-0.065) | | 0.051(0.032-0.075) | 0.044(0.026-0.066) | | 0.012(0.007-0.017) | | | 0.183(0.111-0.276) | |
| Kyrgyz Republic | 6.411(3.932-9.468) | 9.005(5.353-13.823) | | 10.393(6.433-15.279) | 8.458(5.038-12.867) | | 2.008(1.272-2.942) | | | 36.275(22.027-54.379) | |
| Lao PDR | 23.856  (14.584-36.915) | 30.750  (18.038-47.061) | | 32.994  (20.478-48.381) | 23.977  (13.977-35.647) | | 5.803  (3.691-8.512) | | | 117.380  (70.768-176.516) | |
| Lesotho | 4.564(3-6.701) | 5.265(3.251-8.009) | | 5.459(3.528-7.924) | 3.965(2.49-5.762) | | 0.962(0.628-1.379) | | | 20.215(12.897-29.775) | |
| Mauritania | 5.142(3.239-7.618) | 6.194(3.746-9.283) | | 5.55(3.44-7.94) | 3.506(2.084-5.102) | | 0.778(0.485-1.149) | | | 21.17(12.994-31.091) | |
| Mongolia | 14.546(8.927-22.317) | 19.506(11.564-30.23) | | 22.17(14.002-31.77) | 16.77(9.967-25.185) | | 3.727(2.391-5.418) | | | 76.719(46.851-114.919) | |
| Morocco | 30.041  (17.618-48.487) | 39.171  (22.097-62.374) | | 44.711  (27.235-66.959) | 36.136  (20.879-54.844) | | 9.592  (5.988-14.058) | | | 159.650  (93.816-246.722) | |
| Myanmar | 56.269  (34.825-85.607) | 77.243  (45.213-121.899) | | 87.643  (54.759-126.785) | 66.229  (40.59-98.423) | | 16.908  (10.815-24.27) | | | 304.294  (186.201-456.984) | |
| Nepal | 391.755  (287.54-498.632) | 526.716  (371.73-708.519) | | 436.474  (328.08-557.205) | 232.752  (170.401-289.993) | | 44.664  (32.73-55.426) | | | 1632.361  (1190.482-2109.775) | |
| Nicaragua | 12.179(7.584-19.405) | 15.173(9.009-24.179) | | 16.227(10.217-23.531) | 11.781(7.024-17.479) | | 2.943(1.88-4.287) | | | 58.303(35.715-88.881) | |
| Nigeria | 465.176  (295.572-686.17) | 596.129  (365.666-911.097) | | 577.498  (372.789-823.844) | 367.222  (225.974-538.73) | | 77.026  (50.025-110.776) | | | 2083.052  (1310.026-3070.617) | |
| Pakistan | 274.190  (176.927-401.968) | 340.542  (212.49-510.457) | | 303.061  (198.852-425.611) | 183.746  (120.073-249.329) | | 39.902  (27.38-53.372) | | | 1141.442  (735.722-1640.737) | |
| Papua New Guinea | 11.574(6.997-17.757) | 14.647(8.611-22.872) | | 15.106(9.527-22.027) | 10.279(5.967-15.418) | | 2.409(1.506-3.507) | | | 54.015(32.609-81.581) | |
| Philippines | 168.186  (101.721-261.542) | 223.985  (130.542-344.79) | | 256.064  (157.534-368.556) | 192.915  (112.111-288.163) | | 52.925  (32.985-77.031) | | | 894.075  (534.893-1340.083) | |
| Samoa | 0.269(0.162-0.405) | 0.405(0.235-0.619) | | 0.459(0.294-0.67) | 0.352(0.21-0.53) | | 0.087(0.055-0.128) | | | 1.573(0.956-2.352) | |
| São Tomé and Principe | 0.525(0.336-0.775) | 0.622(0.374-0.942) | | 0.529(0.335-0.771) | 0.33(0.2-0.491) | | 0.068(0.042-0.1) | | | 2.073(1.288-3.078) | |
| Senegal | 16.501(10.577-24.879) | 19.567(11.908-30.478) | | 18.077(11.222-26.599) | 11.795(7.254-17.435) | | 2.563(1.619-3.672) | | | 68.503(42.581-103.062) | |
| Solomon Islands | 1.512(0.905-2.329) | 1.965(1.132-3.043) | | 1.955(1.218-2.851) | 1.416(0.838-2.101) | | 0.368(0.234-0.536) | | | 7.216(4.328-10.859) | |
| Sri Lanka | 55.975  (34.234-87.057) | 76.898  (45.012-121.605) | | 92.583  (57.913-135.829) | 75.605  (45.487-114.908) | | 20.660  (13.203-30.235) | | | 321.721  (195.848-489.635) | |
| Tajikistan | 2.648(1.602-4.113) | 3.597(2.099-5.692) | | 4.233(2.681-6.158) | 3.344(2.046-5.026) | | 0.75(0.488-1.103) | | | 14.572(8.915-22.092) | |
| Tanzania | 383.081  (241.588-609.723) | 498.375  (309.736-772.073) | | 401.779  (276.752-544.811) | 207.372  (148.27-265.871) | | 39.260  (29.397-50.37) | | | 1529.868  (1005.743-2242.848) | |
| Timor-Leste | 1.54(0.935-2.332) | 2.183(1.294-3.421) | | 2.049(1.284-3.017) | 1.393(0.799-2.096) | | 0.476(0.295-0.7) | | | 7.642(4.606-11.566) | |
| Tunisia | 12.190  (7.251-19.386) | 15.035  (8.721-23.996) | | 17.782  (11.166-26.258) | 14.376  (8.299-21.705) | | 3.870  (2.371-5.72) | | | 63.252  (37.809-97.065) | |
| Ukraine | 106.914  (64.747-166.946) | 155.975  (90.549-240.09) | | 193.033  (120.196-278.114) | 195.991  (115.786-293.577) | | 61.790  (39.328-90.35) | | | 713.703  (430.608-1069.076) | |
| Uzbekistan | 48.333  (29.84-72.906) | 63.922  (37.851-98.036) | | 74.480  (47.424-107.261) | 61.740  (37.125-91.397) | | 14.531  (9.322-21.348) | | | 263.006  (161.562-390.948) | |
| Vanuatu | 0.705(0.43-1.095) | 0.914(0.534-1.396) | | 0.966(0.599-1.422) | 0.711(0.419-1.066) | | 0.205(0.128-0.299) | | | 3.501(2.11-5.278) | |
| Vietnam | 408.244  (245.438-630.296) | 574.417  (334.012-918.985) | | 651.654  (420.177-952.141) | 511.939  (315.885-766.441) | | 126.305  (80.59-186.576) | | | 2272.558  (1396.1-3454.438) | |
| Zambia | 34.167(21.546-51.894) | 39.839(23.89-60.808) | | 35.327(22.299-51.437) | 21.935(12.713-32.65) | | 4.958(3.113-7.272) | | | 136.225(83.561-204.061) | |
| Zimbabwe | 96.860  (61.778-143.695) | 106.141  (64.443-164.95) | | 84.146  (53.613-123.133) | 51.620  (31.041-74.834) | | 12.345  (8.002-17.711) | | | 351.111  (218.876-524.323) | |
| Albania | 12.315  (7.528-19.148) | 20.455  (12.351-31.951) | | 28.184  (17.786-40.879) | 25.753  (14.77-38.524) | | 7.280  (4.484-10.643) | | | 93.987  (56.920-141.145) | |
| Argentina | 39.798  (21.363-65.735) | 46.055  (23.878-75.453) | | 47.266  (26.07-73.101) | 37.193  (19.159-59.225) | | 11.030  (6.303-17.491) | | | 181.342  (96.773-291.004) | |
| Armenia | 8.069  (4.946-12.292) | 10.559  (6.138-16.418) | | 13.644  (8.518-19.806) | 14.637  (8.723-22.503) | | 4.399  (2.734-6.483) | | | 51.308  (31.058-77.503) | |
| Azerbaijan | 51.049  (31.346-77.504) | 73.810  (43.497-115.124) | | 99.463  (62.193-144.159) | 87.536  (52.802-129.57) | | 20.598  (13.317-29.843) | | | 332.456  (203.155-496.200) | |
| Belarus | 95.362  (59.552-142.337) | 141.662  (84.592-214.559) | | 177.576  (111.882-257.989) | 178.499  (106.794-271.059) | | 51.901  (33.584-76.112) | | | 645.001  (396.404-962.056) | |
| Bosnia and Herzegovina | 11.911  (7.119-18.221) | 16.877  (9.968-26.139) | | 22.465  (14.126-32.592) | 20.787  (11.989-30.954) | | 6.298  (3.929-9.290) | | | 78.338  (47.132-117.195) | |
| Botswana | 63.513  (40.789-94.405) | 70.324  (43.759-108.939) | | 58.246  (37.194-84.956) | 37.806  (23.021-55.14) | | 8.310  (5.311-11.995) | | | 238.200  (150.073-355.435) | |
| Brazil | 918.610  (554.209-1413.595) | 1189.782  (693.319-1862.06) | | 1447.97  (884.94-2096.458) | 1159.035  (676.994-1743.632) | | 323.304  (201.509-466.027) | | | 5038.701  (3010.971-7581.772) | |
| Bulgaria | 59.145  (35.681-90.962) | 87.601  (51.647-134.642) | | 104.011  (65.196-152.408) | 92.190  (54.905-136.171) | | 30.385  (19.336-43.837) | | | 373.333  (226.765-558.020) | |
| China | 32583.682  (21107.236  -48231.671) | 59874.010  (37230.912  -91249.217) | | 72121.997  (47017.312  -103000.743) | 44434.243  (27552.580  -64684.421) | | 10343.406  (6732.225  -14861.196) | | | 219357.339  (139640.265  -322027.248) | |
| Colombia | 282.791  (169.155-426.66) | 397.492  (234.084-613.033) | | 493.699  (307.204-714.014) | 383.061  (225.067-574.787) | | 97.043  (61.784-141.063) | | | 1654.085  (997.293-2469.558) | |
| Costa Rica | 59.518  (35.755-90.693) | 78.855  (46.707-120.251) | | 99.091  (62.800-143.085) | 79.273  (48.111-119.044) | | 20.265  (13.21-29.834) | | | 337.003  (206.583-502.907) | |
| Cuba | 72.459  (44.006-109.532) | 144.713  (83.234-222.306) | | 178.370  (114.172-258.303) | 128.183  (79.223-190.867) | | 25.992  (16.939-37.927) | | | 549.717  (337.574-818.935) | |
| Dominican Republic | 89.656  (55.594-136.331) | 116.514  (68.896-179.151) | | 120.937  (76.304-177.293) | 83.748  (50.416-127.152) | | 20.066  (12.736-29.431) | | | 430.921  (263.947-649.358) | |
| Ecuador | 85.097  (50.69-129.796) | 110.216  (65.196-171.911) | | 119.780  (72.613-174.780) | 91.876  (53.611-138.484) | | 23.120  (14.206-33.217) | | | 430.089  (256.315-648.189) | |
| Equatorial Guinea | 8.47(5.382-12.629) | 9.857(5.862-15.422) | | 9.271(5.703-13.664) | 5.905(3.422-8.969) | | 1.357(0.851-1.996) | | | 34.86(21.22-52.68) | |
| Fiji | 3.408(2.061-5.269) | 4.502(2.652-6.948) | | 5.626(3.444-8.29) | 4.609(2.674-7.037) | | 1.152(0.723-1.697) | | | 19.297(11.554-29.24) | |
| Gabon | 8.807(5.458-13.526) | 11.367(6.797-17.387) | | 11.631(7.314-16.695) | 8.158(4.912-12.063) | | 1.887(1.202-2.73) | | | 41.849(25.684-62.401) | |
| Georgia | 16.026  (9.565-25.352) | 23.609  (13.998-37.26) | | 30.093  (18.552-44.314) | 29.913  (17.26-45.039) | | 9.014  (5.613-13.069) | | | 108.656  (64.988-165.033) | |
| Guatemala | 57.547  (35.735-91.718) | 66.873  (39.704-106.846) | | 68.434  (43.106-99.306) | 51.248  (30.564-76.074) | | 13.590  (8.685-19.795) | | | 257.692  (157.795-393.738) | |
| Guyana | 2.703(1.657-4.188) | 3.672(2.112-5.851) | | 4.025(2.494-5.973) | 3.019(1.766-4.548) | | 0.744(0.466-1.104) | | | 14.163(8.495-21.665) | |
| Iraq | 43.283  (25.969-66.939) | 57.831  (33.246-90.194) | | 54.499  (33.819-80.941) | 36.556  (21.969-55.428) | | 9.260  (5.912-13.527) | | | 201.430  (120.916-307.028) | |
| Jamaica | 15.012(9.227-23.199) | 21.585(12.756-33.265) | | 23.647(15.001-34.457) | 17.226(10.51-25.59) | | 4.235(2.766-6.258) | | | 81.704(50.261-122.769) | |
| Jordan | 8.096(4.742-12.589) | 10.532(6.001-16.5) | | 10.946(6.644-16.152) | 7.002(3.991-10.885) | | 1.517(0.936-2.246) | | | 38.092(22.314-58.372) | |
| Kazakhstan | 199.973  (121.67-304.616) | 278.150  (162.696-427.883) | | 319.851  (197.252-466.265) | 278.123  (165.325-412.511) | | 70.912  (44.824-103.043) | | | 1147.009  (691.767-1714.317) | |
| Lebanon | 10.329(6.214-16.043) | 14.061(8.115-21.886) | | 15.204(9.535-22.293) | 11.94(7.335-18.158) | | 2.97(1.894-4.374) | | | 54.504(33.093-82.755) | |
| Libya | 17.921(10.567-27.881) | 22.053(12.793-34.703) | | 21.032(13.121-30.593) | 12.862(7.468-19.314) | | 2.943(1.829-4.283) | | | 76.811(45.779-116.774) | |
| Malaysia | 312.237  (185.785-474.236) | 399.353  (232.753-619.61) | | 454.145  (286.654-661.84) | 352.982  (205.34-528.277) | | 93.470  (57.842-136.507) | | | 1612.188  (968.374-2420.47) | |
| Maldives | 7.321(4.452-11.36) | 8.098(4.632-12.748) | | 7.728(4.713-11.25) | 5.196(2.943-7.78) | | 1.149(0.725-1.649) | | | 29.491(17.464-44.787) | |
| Mauritius | 11.426  (7.015-17.518) | 15.276  (8.929-23.852) | | 21.019  (12.984-31.024) | 18.096  (10.868-27.375) | | 4.827  (3.060-7.113) | | | 70.643  (42.856-106.883) | |
| Mexico | 811.566  (498.5-1245.965) | 1143.776  (677.899-1764.333) | | 1304.110  (810.437-1885.345) | 986.317  (578.547-1474.592) | | 270.090  (169.564-391.734) | | | 4515.858  (2734.947-6761.969) | |
| Moldova | 12.073(7.168-18.092) | 16.51(9.742-25.285) | | 19.688(12.351-28.581) | 19.65(11.782-29.453) | | 5.534(3.477-8.117) | | | 73.454(44.52-109.528) | |
| Montenegro | 3.918(2.371-5.996) | 5.582(3.189-8.709) | | 7.074(4.428-10.433) | 6.400(3.950-9.526) | | 1.956(1.247-2.784) | | | 24.929(15.184-37.448) | |
| Namibia | 28.384  (18.37-41.866) | 33.826  (20.652-51.974) | | 29.123  (18.922-42.822) | 17.416  (10.897-25.578) | | 3.723  (2.398-5.349) | | | 112.472  (71.238-167.59) | |
| North Macedonia | 9.244(5.735-14.056) | 13.46(8.055-20.657) | | 17.007(10.886-24.557) | 14.337(8.76-21.087) | | 4.275(2.787-6.292) | | | 58.323(36.222-86.649) | |
| Panama | 60.657  (36.386-91.664) | 86.041  (50.076-134.024) | | 97.031  (61.569-141.95) | 71.162  (42.906-105.95) | | 17.908  (11.458-25.967) | | | 332.800  (202.394-499.555) | |
| Paraguay | 33.055(19.617-51.579) | 41.244(23.806-65.162) | | 46.117(28.192-65.91) | 35.55(21.261-53.216) | | 9.14(5.739-13.183) | | | 165.106(98.615-249.05) | |
| Peru | 255.020  (153.85-391.82) | 331.554  (193.64-524.719) | | 363.384  (227.044-532.068) | 262.872  (155.354-399.145) | | 66.400  (42.082-98.398) | | | 1279.229  (771.97-1946.15) | |
| Romania | 230.469  (140.76-354.651) | 334.767  (193.257-523.019) | | 404.518  (261.638-592.334) | 278.604  (171.408-416.196) | | 112.842  (72.951-163.775) | | | 1361.200  (840.014-2049.975) | |
| Russian Federation | 2195.473  (1379.723-3322.08) | 3167.937  (1912.863-4838.15) | | 3688.671  (2391.065-5276.248) | 3946.309  (2406.914-5828.11) | | 1195.156(772.289-1728.274) | | | 14193.546(8862.853-20992.861) | |
| Serbia | 50.178  (30.359-77.12) | 70.627  (41.045-108.779) | | 85.926  (53.775-123.327) | 73.635  (44.023-109.559) | | 26.575  (17.081-38.938) | | | 306.941  (186.282-457.722) | |
| South Africa | 911.801  (622.273-1293.008) | 1116.842  (716.047-1644.932) | | 989.564  (664.89-1394.004) | 621.164  (398.185-871.826) | | 137.359  (93.249-189.367) | | | 3776.731  (2494.644-5393.136) | |
| St. Lucia | 2.642(1.595-4.046) | 3.916(2.251-6.057) | | 4.436(2.749-6.425) | 3.071(1.84-4.546) | | 0.718(0.453-1.062) | | | 14.783(8.889-22.137) | |
| St. Vincent and the Grenadines | 0.818(0.515-1.245) | 1.164(0.689-1.816) | | 1.351(0.841-1.962) | 1.054(0.623-1.57) | | 0.271(0.171-0.403) | | | 4.658(2.84-6.997) | |
| Suriname | 2.325(1.441-3.603) | 3.476(2.069-5.289) | | 4.128(2.594-6.08) | 2.971(1.768-4.414) | | 0.678(0.427-1.002) | | | 13.577(8.3-20.389) | |
| Thailand | 721.841  (451.978-1109.206) | 1111.508  (652.843-1732.64) | | 1376.214  (841.584-2000.096) | 1083.370  (629.927-1606.124) | | 284.744  (181.433-411.078) | | | 4577.676  (2757.765-6859.143) | |
| Tonga | 0.191(0.118-0.292) | 0.27(0.159-0.416) | | 0.311(0.196-0.449) | 0.218(0.136-0.323) | | 0.056(0.035-0.081) | | | 1.046(0.645-1.562) | |
| Turkey | 284.600  (172.738-446.157) | 345.663  (199.941-543.986) | | 396.098  (242.336-579.531) | 308.537  (178.681-463.194) | | 81.749  (51.949-119.137) | | | 1416.646  (845.645-2152.004) | |
| Turkmenistan | 28.769  (17.649-43.843) | 39.536  (23.194-61.18) | | 46.031  (29.454-65.669) | 38.299  (23.289-57.415) | | 9.251  (5.882-13.406) | | | 161.885  (99.469-241.514) | |
| TOTAL | 52042.478  (33420.486  -77393.212) | 85330.309  (52635.905  -130212.801) | | 98316.055  (63728.356  -140809.165) | 64066.318  (39447.275  -93587.463) | | 15446.336  (10007.082  -22238.219) | | | 315201.495  (199239.104  -464240.861) | |
| Captions：Location according to the GBD 2019 study of Low-and Middle-Income Countries. The productivity loss of presbyopia are given inform with 95% confidence interval. | | | | | | | | | | | |
